# A Comprehensive tRNA Deletion Library Unravels the Genetic Architecture of the tRNA Pool

**DOI:** 10.1371/journal.pgen.1004084

**Published:** 2014-01-16

**Authors:** Zohar Bloom-Ackermann, Sivan Navon, Hila Gingold, Ruth Towers, Yitzhak Pilpel, Orna Dahan

**Affiliations:** Department of Molecular Genetics, Weizmann Institute of Science, Rehovot, Israel; The University of North Carolina at Chapel Hill, United States of America

## Abstract

Deciphering the architecture of the tRNA pool is a prime challenge in translation research, as tRNAs govern the efficiency and accuracy of the process. Towards this challenge, we created a systematic tRNA deletion library in *Saccharomyces cerevisiae*, aimed at dissecting the specific contribution of each tRNA gene to the tRNA pool and to the cell's fitness. By harnessing this resource, we observed that the majority of tRNA deletions show no appreciable phenotype in rich medium, yet under more challenging conditions, additional phenotypes were observed. Robustness to tRNA gene deletion was often facilitated through extensive backup compensation within and between tRNA families. Interestingly, we found that within tRNA families, genes carrying identical anti-codons can contribute differently to the cellular fitness, suggesting the importance of the genomic surrounding to tRNA expression. Characterization of the transcriptome response to deletions of tRNA genes exposed two disparate patterns: in single-copy families, deletions elicited a stress response; in deletions of genes from multi-copy families, expression of the translation machinery increased. Our results uncover the complex architecture of the tRNA pool and pave the way towards complete understanding of their role in cell physiology.

## Author Summary

Transfer RNAs are an important component of the translation machinery. Despite extensive biochemical investigations, a systems-level investigation of tRNAs' functional roles in physiology, and genetic interactions among them, is lacking. We created a comprehensive tRNA deletion library in yeast and assessed the essentiality of each tRNA in multiple conditions. The majority of tRNA deletions showed no appreciable fitness defect when such strains were grown on rich medium. More challenging environmental conditions, however, revealed a richer set of specific-tRNA phenotypic defects. Co-deletion of tRNA combinations revealed that tRNAs with essential function can be compensated by members of the same or different anti-codon families. We often saw that identical tRNA gene copies contribute deferentially to fitness, suggesting that the genomic context of each gene can affect functionality. Genome-wide expression changes in response to tRNA deletions revealed two different responses. When a deleted tRNA belongs to a family which contains multiple genes with the same anti-codon, the affected cells responded by up-regulating the translation machinery, but upon deletion of singleton tRNAs, the cellular response resembled that of proteotoxic stress. Our tRNA deletion library is a unique resource that paves the way towards fully characterizing the tRNA pool and their role in cell physiology.

## Introduction

Messenger RNA translation is a central molecular process in any living cell and is among the most complicated and highly regulated of cellular processes [1], [2]. The tRNA pool is a fundamental component in that process, serving as the physical link between the nucleotide sequence of mRNAs and the amino acid sequence of proteins. In the cycle of translation elongation, tRNA selection is considered the rate-limiting step [3], therefore tRNA availability is one of the major factors that govern translation-efficiency and accuracy of genes [4], [5].

Previous studies have established that efficient translation can increase protein levels and provide a global fitness benefit by elevating the cellular concentrations of free ribosomes [6], [7], while accurate translation benefits the cell by reducing the metabolic cost of mis-incorporation events [8].

The tRNA pool is composed of various tRNA isoacceptor families, each family carries a different anti-codon sequence that decodes the relevant codon by Watson-Crick base pairing, or codons with non-perfect base pairing of the third nucleotide by the wobble interaction. tRNA families are further classified to isotypes if they carry the same amino acid. In all eukaryotic genomes, each tRNA family can be encoded by a single or multiple gene copies [9], [10]. It was previously shown for several organisms that the concentrations of various tRNA isoacceptors positively correlates with the tRNA family's gene copy-number [11], [12]. These observations along with detailed analysis of the relationship between gene copy-number of tRNA families and codon-usage, established the notion that the multiplicity of tRNA genes in yeast is not functionally redundant. Such multiplicity might establish the correct balance between tRNA concentrations and the codon usage in protein-coding genes [13], thus justifying the use of the tRNA gene copy-number as a proxy for actual tRNA amounts [12], [14], [15].

The transcription of tRNA genes is catalyzed by RNA polymerase III (pol III), promoted by highly conserved sequence elements located within the transcribed region [16]. A genome wide analysis of pol III occupancy in yeast revealed that virtually all tRNA genes are occupied by the pol III machinery [17]–[19], and are thus considered to be genuinely transcribed. This observation, combined with the fact that tRNA genes within a family are highly similar, led to the notion that all copies within a family contribute equally to the total expression level and hence to the tRNA pool.

Although tRNAs have been extensively studied, until very recently many of the studies were performed on individual genes at the biochemical level. Only in recent years systematic genome-wide approaches started to complement the biochemical approach. These studies reveal a much more complex picture in which pol III occupancy, a proxy for tRNA transcription, varies within families and between tissues [17], [20]–[22]. Expression however does not equal function, and so far no systematic study has been carried out to decipher the specific contribution of each tRNA gene to the tRNA pool and to the cell's fitness.

To study the role of individual tRNAs and the architecture of the entire tRNA pool, we created a comprehensive tRNA deletion library in the yeast *Saccharomyces cerevisiae*. The library includes 204 deletions of nuclear-encoded tRNA genes out of the total 275 present in the yeast genome. In addition, we created double deletions of selected tRNA gene combinations and of specific tRNAs with a tRNA modifying enzyme. We developed a robotic method to screen and score various fitness parameters for these deletion strains, and applied it across various growth conditions. This systematic deletion library revealed an architecture of genetic interactions that feature extensive backup-compensations within and between tRNA families. Such compensation capacity endows the organism with robustness to environmental changes and to genetic mutations. We found that different copies within a tRNA family contribute differently to the organism's fitness, revealing a higher level of complexity in the tRNA pool's architecture, possibly at the regulatory level. Finally, we observed two distinct molecular signatures that underlie the cellular response to changes in the tRNA pool. First, the deletion of non-essential single-copy tRNA genes invoked proteotoxic stress responses, indicating a connection between aberrant tRNA availability and protein misfolding. Second, the deletion of representative tRNAs from multi-copy families triggered milder responses by up-regulating genes that are involved in the translation process. Together our results uncover the complex architecture of the tRNA pool reviling a profound effect on cellular fitness and physiology.

## Results

### Generation of a tRNA deletion library in *S. cerevisiae*

**Figure 1:**
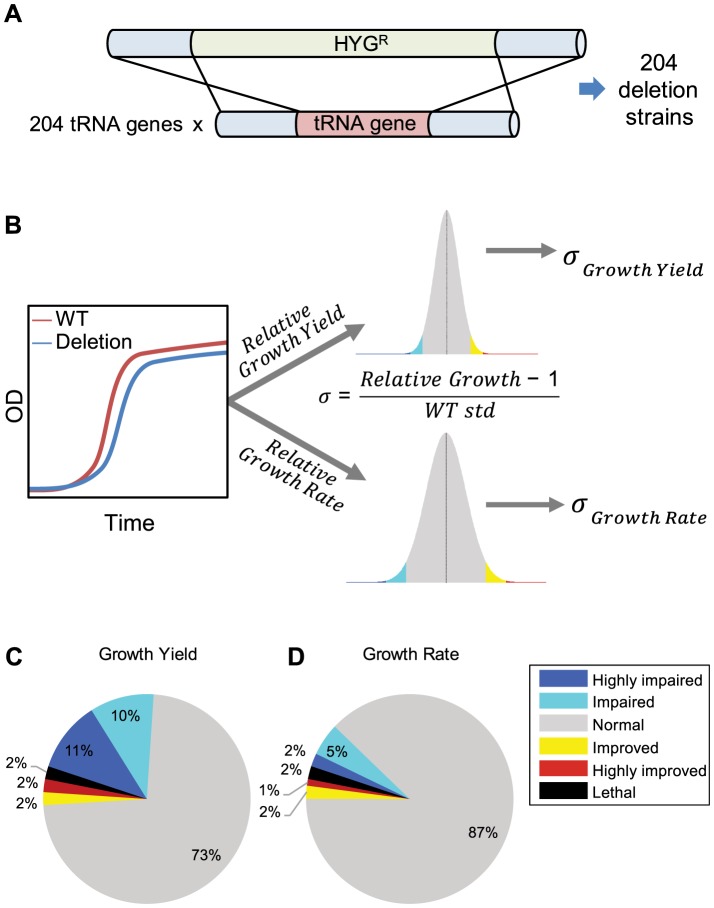
Creation and analysis of tRNA deletion library. (A) Schematic representation of the deletion process. 204 different tRNA strains were created using homologous recombination. In each strain, a different tRNA gene was replaced by a hygromycin B resistance marker. (B) Schematic representation of growth measurements, analysis, and scoring. For each strain, relative-growth-rate and relative-growth-yield are calculated in relation to the wild-type strain. These parameters are then projected on a distribution of the wild-type growth parameters. Sigma (σ) is calculated according to the formula and denotes the number of standard deviations from the mean of the wild-type (see also Supplemental figure S1A). The color in the histogram are areas were: σ<−3 (blue), −3<σ<−2 (cyan), 2<σ<3 (yellow) and 3<σ (red). The same color code is used to define phenotypes in the pie charts (C and D). (C–D) Distribution of phenotypes for the tRNA deletion library in rich medium, according to two growth parameters: relative growth yield (C) relative growth rate (D). Deletion strains were assigned to categories according to their σ values. Any absolute σ value larger than 2 was considered as non-normal phenotype, where negative sigma denotes impairment (worse than the wild-type) and positive sigma denotes improvement (better than the wild-type). Any absolute σ value larger than 3 was considered as a strong phenotype. Thus, highly impaired for σ<−3, impaired for −2>σ>−3, improved for 2<σ<3, and highly improved for σ>3, see also Supplemental figure S1B.

To gain a better understanding of the functional role of individual tRNA genes and their contribution to the tRNA pool, we created a comprehensive tRNA deletion library in *S. cerevisiae*, where in each strain a single nuclear–encoded tRNA gene was deleted. This methodology is based on recombining a selective marker into the genome at the expense of the deleted gene, as was done in the creation of the yeast ORF deletion library [23] (Figure 1A). A particular challenge in targeting specific tRNA genes for deletion by such a method stems from the high degree of sequence similarity within tRNA families, which can share 100% sequence identity. Consequently, in order to create specific gene deletions, we relied on unique sequences that overlap or flank the tRNA genes (see Supplemental text S1). Our tRNA deletion library contained 204 deletions out of the 275 nuclear-encoded tRNA genes identified in *S. cerevisiae* (see Materials and Methods). These deletions covered all 20 amino acids and 40 of the 42 anti-codon families. The remaining 71 tRNA genes were not deleted due to their complex genomic surrounding, since such deletions might affect neighboring potential features in their genomic vicinities. The library also consisted of 50 strains that represent various combinations of tRNA deletions, and co-deletions of selected tRNAs with the *TRM9* gene which codes for an enzyme that post-transcriptionally modifies tRNA molecules.

Although the majority of tRNA families contain multiple gene copies, there are six single-copy tRNA families in the *S. cerevisiae* genome. Out of these singleton families, four (*tS(CGA)*, *tR(CCG)*, *tQ(CAG) tT(CGU)*) were found to be essential in our analysis, which confirms previous reports [24]–[26](see Supplemental text S1). The remaining two singleton families (*tR(CCU)*, *tL(GAG)*) were identified as non-essential upon deletion. All the tRNA genes that belong to multi-copy families were non-essential upon deletion.

### Cells were robust to tRNA gene deletions in rich medium but reveal sensitivity in challenging conditions

To assess the contribution of each tRNA gene to cellular growth, we attempted to accurately characterize the growth dynamics of each deletion strain by implementing a robotic method to screen and score growth phenotypes of all tRNA deletion strains in a given growth condition. This fitness measurement approach allowed us to differentiate between physiological effects of the deletion under different growth phases, unlike the competition approaches for fitness measurement [27] that typically integrated all growth phases. We characterized each deletion strain by two growth parameters: growth rate and growth yield, the latter is defined as the size of the population upon entering stationary phase (Figure 1B and Supplemental figure S1A).

We began the characterization of the tRNA deletion library by growing the strains in rich medium. Under this condition, 13% of the deletion strains demonstrated a phenotype in growth rate and 27% showed a growth yield phenotype (Figure 1C–D and Supplemental figure S1B). Most strains exhibited a notable phenotype only in one of the two parameters. Strains that showed altered phenotypes in both growth rate and yield were rare (Supplemental figure S1B). Overall, most tRNA deletion strains did not exhibit any altered growth phenotype in rich medium, indicating robustness to tRNA gene deletion. Seven percent of the tRNA deletion strains resulted in growth improvement, suggesting that for some genes the cost of retaining them in the genome and/or expressing them may exceed their benefit in this condition. Similar observations were also made on a selection of protein-coding genes in this species [28]. Apart from the singletons whose deletion strains were often dead or exhibit impaired growth, we could not explain the observed growth phenotypes in growth rate or yield by either tRNA family size or amino acid identity (Supplemental figure S2). To further examine the phenotypes of the tRNA deletion strains, we calculated the correlation to the mRNA expression level of adjacent genes and found none (see, Supplemental table S1 figure S3 and Supplemental text S1).

Given that yeast cells are constantly exposed to varying environmental conditions, their tRNA repertoire should differentially accommodate growth in various environments. We next examine whether stressful conditions would retain the robustness observed in rich medium or reveal another set of condition-dependent growth phenotypes. We screened the deletion library under a diverse set of growth conditions including different metabolic challenges and stress-inducing reagents reported in previous studies [29]–[31]. The fact that the production of tRNA molecules is considered energetically costly [32] prompted us to explore the effect of carbon limitation, alternative carbon sources and minimal medium on tRNA essentiality.

**Figure 2:**
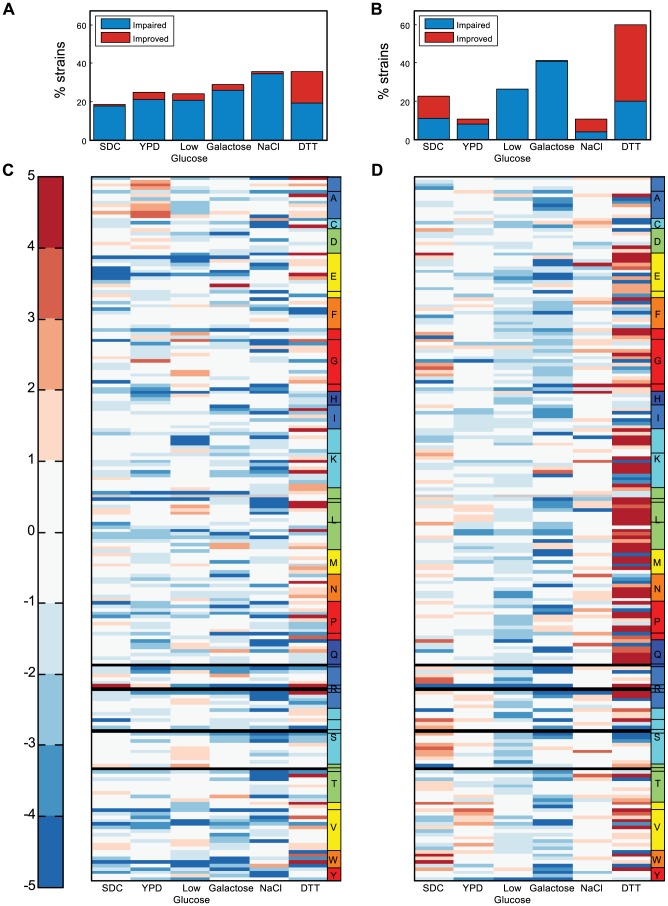
Screening the tRNA deletion library across various growth conditions. (A) Percent of strains exhibiting a growth yield phenotype in various conditions. The color indicates the type of phenotype: impaired (blue) or improved (red). (B) Percent of strains exhibiting a growth rate phenotype in various conditions. (C–D) The σ values measured for both the growth yield (C) and the growth rate (D) for all deletion strains across six conditions. The color bar indicates the σ values, red denoting improvement and blue impairment. Each row denotes a tRNA deletion strain and each column denotes different growth condition. Strains are ordered on the y-axis according to amino acids (denoted by letter) and further separated into families (denoted by lines within the amino-acid box). Black rows denote lethal strains. Gray rows indicate strains for which the respective value was not measured.

Growing the tRNA deletion library under stressful conditions revealed condition-specific phenotypes (Figure 2A–D, Supplemental table S2). In all but one of the examined conditions (Dithiothreitol-DTT, a reducing agent that also inflicts a general protein-unfolding stress), robustness to tRNA gene deletion was maintained. In the DTT condition, the phenotypes were surprising: while multiple tRNA deletions exhibited impaired growth rates, many also demonstrated growth rate improvements (Figure 2B, 2D). As in the rich medium condition, we could not explain the observed growth phenotypes by either the family size, or the amino acid identity in all of the examined stress conditions.

### Extensive redundancy underlies robustness to tRNA gene deletion

**Figure 3:**
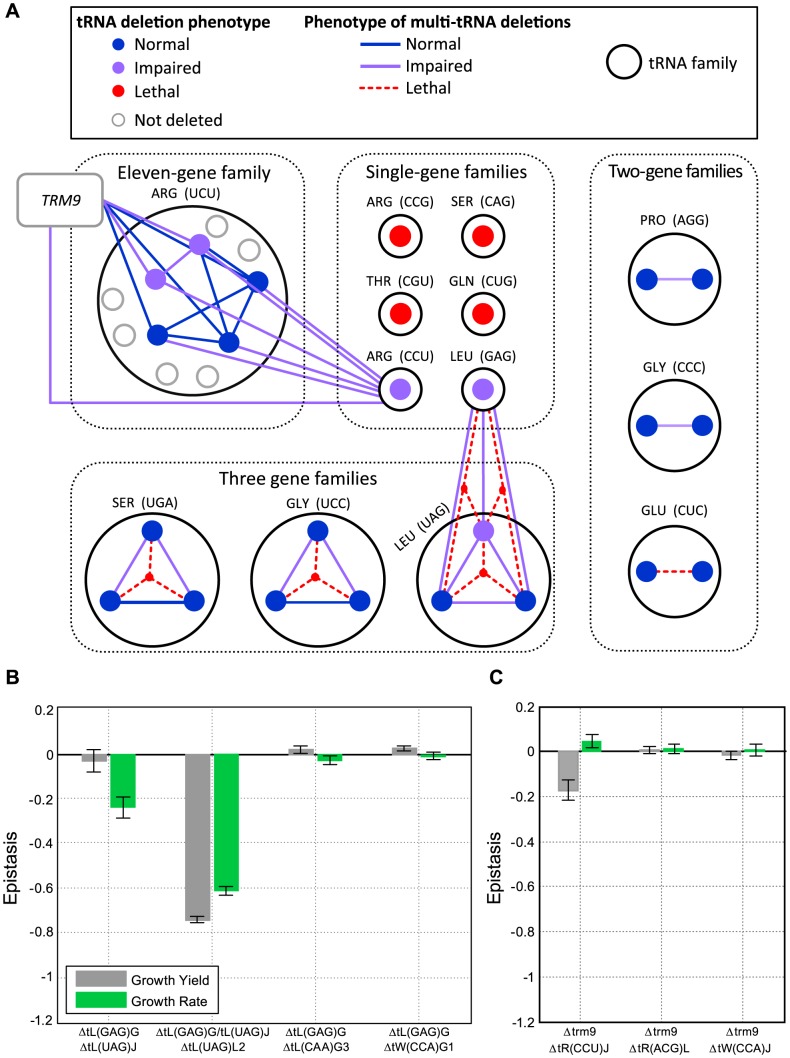
Extensive redundancy underlies robustness to tRNA gene deletion. (A) Schematic representation of the genetic interactions within and between tRNA families. Families are denoted by dark grey circles and grouped (black dashed line) according to their tRNA copy number. Each family is denoted by its anti-codon and amino-acid. A protein-coding gene i.e. *TRM9* is denoted by a grey box. Each filled circle indicates a tRNA deletion strain. The lines connecting the deletion strains denote a co-deletion of these genes (a multi-tRNAs deletion strain). The color of the filled circles and lines denote the severity of the growth phenotype for the respective strain: blue for normal growth, purple for impaired growth (worse than wild-type) and red for lethality. (B) Epistasis values for multi-tRNAs deletion strains which contain the deletion of *tL(GAG)* and either: one *tL(UAG)* gene, two *tL(UAG)* genes, *tL(CAA)* (which is a tRNA of different Leucine family), and *tW(CCA)* (which is a non-Leucine tRNA) as controls. (C) Epistasis values for multi-tRNAs deletion strains which contain the deletion of *trm9* with: the singleton *tR(CCU)*, and *tR(ACG)* which is a tRNA of different Arginine family and *tW(CCA)* which is a non- Arginine tRNA as controls. In both (B) and (C) epistasis values of the relative growth yield and growth rate are indicated in grey and green respectively. Data is presented as mean of 3 biological repetitions +/− SEM.

Our observations of robustness to tRNA gene deletions in rich medium, as well as several stressful growth conditions, prompted us to further explore the genetic architecture conferring this phenotype. Given that most tRNA families contain multiple gene copies, we hypothesized that at least part of the observed robustness might be the outcome of compensation provided by the remaining genes in the family. In addition, due to wobble-interactions, robustness may also be the outcome of compensation between families of the same isotype. Focusing on rich medium conditions, we generated selected combinations of multiple tRNA deletions. To examine the first possibility we created deletions of entire two-member and three-member tRNA families. As shown in figure 3A such family deletions resulted in either lethality (indicating a loss of the family's function), or viability with growth impairment (indicating a partial compensation of the family's function by other families).

We then turned to examine in more detail the interactions within these essential three-gene families by examining the growth of various double deletion strains. Contrary to the common notion that suggests little or no functional redundancy between tRNA gene copies [13], we observed that in each of these families any one family member can sustain normal or near-normal fitness (Figure 3A, Supplemental figure S4A–B and Supplemental table S3). Similar observations were made for essential two-gene families upon one member's deletion (Figure 3A). Such results can either imply that yeast cells carry more tRNA copies than are actually needed to sustain growth under optimal growth conditions, or that a responsive backup mechanism might be at work, one that provides compensation by increasing the transcription of the remaining copies, as was previously observed in protein-coding genes [33]–[35]. We thus decided to investigate the expression levels of certain tRNA families, using RT-qPCR (Figure S5). For each deletion, we compared the expression level of the remaining copies belonging to the designated family to that of a wild-type strain. We observed in most strains an expected reduction in expression of the respective family. These findings suggest that in these families, tRNA supply exceeds the demand under rich medium conditions (Figure S5A). However in some cases there were no such decreases in expression, there were even observable increases, demonstrating that a responsive backup mechanism may have been at work, inducing the expression of the remaining family members following deletion of a certain member (Figure S5B).

Next, we turned to examine the surprising cases in which the deletion of an entire tRNA gene family resulted in a viable strain. We reasoned that in these cases a different type of compensation, which is based on wobble interactions across iso-acceptor families, came into play. To decipher this compensation mechanism we focused on the genetic interactions involving the two non-essential singleton families, *tL(GAG)* and *tR(CCU)* (Figure 3A).

In the absence of *tL(GAG)*, the members of the *tL(UAG)* family represent the sole tRNA that can decode the CUN Leucine codons, and might be a candidate for providing compensation upon deletion of *tL(GAG)* even though such decoding does not match the classic wobble rules [36]. Co-deletion of *tL(GAG)* with one of the *tL(UAG)* gene copies resulted in growth aggravation and negative epistasis. Deletion of the *tL(GAG)* together with two copies of the *tL(UAG)* family was lethal despite the fact that one copy of *tL(UAG)* still remained in the genome, indicating that a single *tL(UAG)* gene was insufficient to compensate for the loss of *tL(GAG)* (Figure 3B). The genetic interaction between *tL(UAG)* and *tL(GAG)* appeared specific, since co-deleting one copy of the *tL(UAG)* family together with two additional tRNA genes (*tL(CAA)G3* and *tW(CCA)G1)* did not generate observable epistasis in either case (Figure 3B). We thus concluded that the *tL(UAG)* family is partially redundant to the *tL(GAG)* family, yet such redundancy was not sufficient to completely compensate for the loss of *tL(GAG)*.

Similarly, the viability of the *tR(CCU)* deletion strain could be due to compensation provided by the 11 copies of the *tR(UCU)* family. Indeed the wobble rules are consistent with this assumption, but such interaction was never functionally demonstrated. Formally, demonstrating that the *tR(UCU)* family can compensate for the loss of the singleton *tR(CCU)* would amount to co-deleting all 12 tRNA genes. Looking for simpler means, we decided on a more economic, albeit indirect way. We co-deleted the singleton *tR(CCU)* with the Trm9 enzyme, which is responsible for methylating the third anticodon position of *tR(UCU)* and *tE(UUC)* [37]. It was previously shown that such methylation is needed for supporting the wobble interaction between *tR(UCU)* tRNAs and the AGG codon (the cognate codon of the CCU anti-codon) [37]. The *tR(CCU)*–*trm9* double deletion strain was viable, but exhibited an appreciable aggravation of growth yield (Figure 3A and 3C). Thus our results confirm that the methylated *tR(UCU)* family can partially compensate for the loss of *tR(CCU)*. We attempted to define a more general role for the Trm9 modification enzyme in modulating the compensation mechanism between tRNA families. To this end we created 10 additional double deletions of the enzyme along with each of 10 tRNA genes from two glutamic acid families, one that is modified by the enzyme and one that is reportedly not modified by the enzyme [38] (see Supplemental figure S6). No epistasis was detected between the enzyme and any of these 10 tRNAs and hence, the data cannot support or exclude a putative similar role of the enzyme beyond the *tR(UCU)* family.

We thus conclude that there are two mechanisms that can account for the observed robustness for tRNA deletions under favorable growth conditions. The first is redundancy within a family, and its efficiency appears to be independent of the number of remaining tRNA gene copies. The second is compensation between families, which operates via wobble interactions.

### Identical tRNA genes contributed differentially to cellular fitness

**Figure 4:**
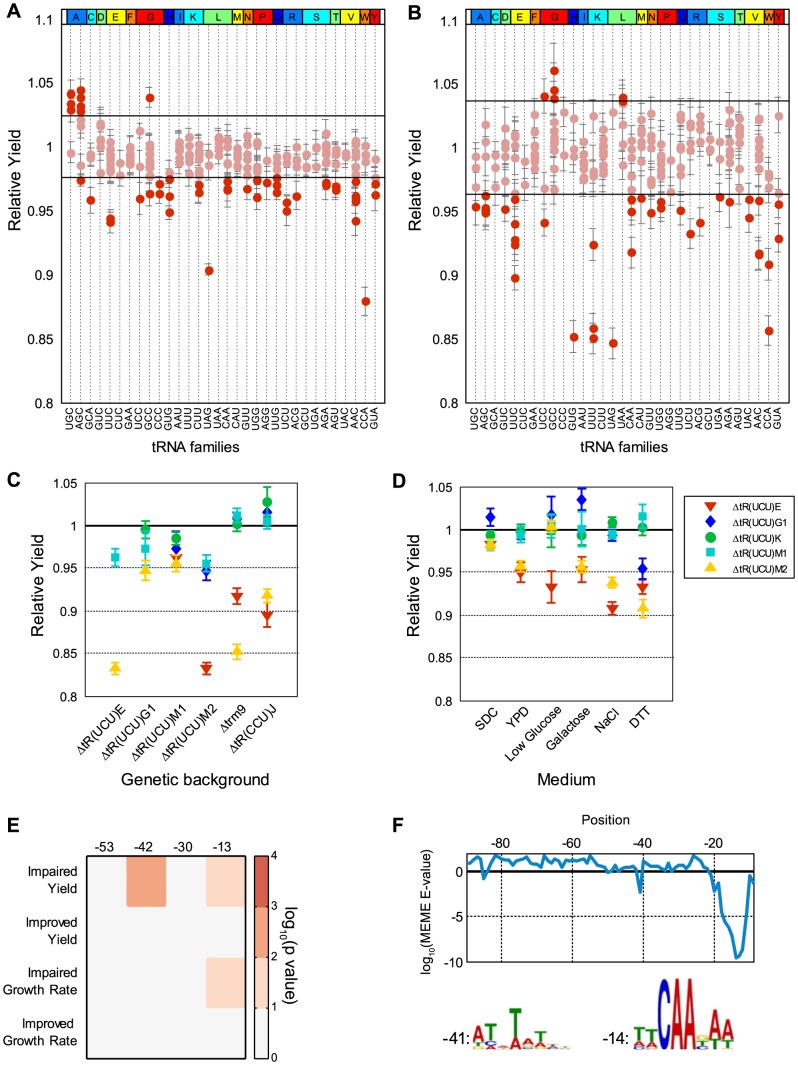
Differential contribution of identical tRNA gene copies. (A–B) Relative growth yield values of the tRNA deletion library strains in rich medium (A) and low glucose (B), sorted by anti-codon and amino-acid identity along the x-axis. Each dot along the vertical lines denotes the value (data are represented as mean of 3 biological repetitions +/− SEM) of a deletion strain of different tRNA gene of the respective family. The horizontal lines mark two standard deviations around the mean of the wild-type. Dots above or below these lines are considered non-normal phenotypes (see also Supplemental figure S7). (C) Relative growth yield values (data are presented as mean of 3 biological repetitions +/− SEM) of various double deletion combinations consisting of: five *tR(UCU)* family members, *tR(CCU)* and *trm9* deletion strains as indicated on the x-axis, along with the five members of the *tR(UCU)* family each denoted by a different shape and color in the legend. (D) Relative growth yield of the five *tR(UCU)* members across different growth conditions, indicated on the x-axis. (E) Enrichment of conserved elements in tRNA genes divided according to phenotype observed in rich media for each growth parameter. Each column in the matrix denotes a conserved element as defined by [42]. Color bar indicates the −log_10_ of the hypergeometric p-value. (F) log10 E-value found by the MEME software for the most significant motif in a 9 bp window starting from the position indicated by the x-axis. The LOGOs of the two significant motifs are displayed below, next to a number indicating its position. Position 0 is the first position of the mature tRNA.

We then asked whether all copies within a family contribute equally to the tRNA pool. It is often implicitly assumed that all tRNA copies contribute similarly to the cellular tRNA pool. However, comparison of the growth parameters of tRNA deletions from the same family revealed marked differences between seemingly identical family members. In particular, under rich medium, 21 out of the 32 deletions examined from multi-copy families showed growth yield differences spanning a broad range of at least 10% (Figure 4A). Such differences were also detected in the growth rate parameter (Supplemental figure S7A) although they were less pronounced. We thus focus on the growth yield parameter in all further analysis. The phenomenon of differential contribution to fitness by different family members was further enhanced when we grew the deletion strains on more challenging conditions such as low glucose (Figure 4B and Supplemental figure S7B). To further investigate the genetic interactions between differentially contributing tRNA copies within a given family, we focused on the *tR(UCU)* family.

The *tR(UCU)* family contains 11 identical copies in the genome, 5 of which were represented in our library. In rich medium, two copies (*tR(UCU)E* and *tR(UCU)M2*) showed appreciable reduction in growth yield (termed Major copies), while deletions of the other three copies (*tR(UCU)M1*, *tR(UCU)G1* and *tR(UCU)K*) grew essentially as the wild-type (termed Minor copies). Introducing a plasmid with the appropriate tRNA gene copy complemented the growth of all deleted copies (Supplemental figure S7C). To further assert the separation between the Major and Minor copies, we examined various pair-wise deletion combinations of these members. All pairs that included at least one Major member exhibited growth impairment upon deletion, while pairs that consisted of only Minor copies demonstrated either a slight growth defect or none at all (Figure 4C). Further analysis of genetic interactions of these family members with either the *TRM9* gene, or with the above mentioned *tR(CCU)* gene that belongs to a different Arginine family, revealed a similar effect (Figure 4C). These results indicate that the loss of different *tR(UCU)* genes in the same genetic background does not affect the phenotype equally, Major copies are more essential than Minor copies and as such are also more essential in providing compensation within the family.

We next turned to examine whether the hierarchy of Major and Minor copies is preserved across various stress conditions (Figure 4D). Examining essentiality in several conditions, we observed the same phenomenon in which Major copies demonstrated a stronger effect on growth compared to Minor copies in most stress conditions. We also noted that the Minor copies showed a diverse response ranging from slight growth improvement, wild-type level growth to observable growth impairment. A potential scenario may be one in which the Major copies always actively contribute to the pool, while the Minor copies might be recruited at times of need to maintain efficient translation. Thus, the loss of a Major copy could only be partially compensated by the remaining copies.

Following these observations, we turned to examine possible genetic elements that might promote the phenomenon of differential contribution. Since all family members have identical sequence, we hypothesized that differential contribution should be due to differences in the vicinity of tRNA genes. To demonstrate this notion we performed a complementation assay, introducing different tRNAs from the UCU family, along with 200 bp of their flanking sequences, to the *tR(UCU)M2* deletion strain. We observed different degrees of complementation. Given that different constructs differ only in the region flanking the tRNA gene, the variation in complementation capability can be attributed to the different sequences flanking the tRNA (Supplemental figure S7D). The effect of sequences that flank tRNA genes on their transcription was reported in multiple studies [39]–[42]. In one such study Giuliodori *et al.* [42] preformed an analysis of conserved sequence elements upstream of *S. cerevisiae* tRNA genes. They identified four conserved sequence elements located at positions −53 (T-rich), −42(TATA-like), −30(T-rich) and −13 (pol III TSS) with respect to the first nucleotide of the mature tRNA. We used these results to examine the entire tRNA deletion library and checked whether tRNA deletions that exhibited or that did not exhibit altered phenotype in rich medium revealed enrichment for any particular motif (Figure 4E). We found that deletions exhibiting phenotypes of growth impairment were significantly enriched for the presence of specific motifs. In particular, deleted strains that exhibited impairment in growth yield had an enrichment for the TATA-like motif at position −42. In addition, the TSS motif at position −13 was enriched in deletion strains that exhibited impairment in both growth rate and yield. To reinforce these observations, we ran the MEME motif search algorithm [43] to screen the upstream sequences of tRNA deleted strains exhibiting impaired growth yield for enriched motifs (see Materials and Methods). Two significant motifs were found that resemble those reported by Giuliodori *et al.* in both sequence and location (Figure 4F).

Together these results indicate that the contribution to the tRNA pool and cellular fitness of different copies of the same tRNA family are far from equal. We provide one possible explanation, which can account for the differential essentiality, implying that the sequences flanking tRNA genes play a role in determining their expression level.

### Physiological effects of tRNA gene deletions on protein folding

As mentioned above, screening the tRNA deletion library in the presence of the reducing agent Dithiothreitol (DTT), a drug that exerts a proteotoxic stress in the cell, showed severe phenotypic defect in many deletion mutants (Figure 2A, B). Yet, many of the strains that demonstrated growth reduction in other conditions were less sensitive than wild-type to this drug (Figure 2C, D). These findings point towards a connection between tRNA functionality and the protein folding state in the cells. To further explore this connection, we turned to thoroughly characterize a selection of tRNA deletions in the presence of various proteotoxic agents. We chose two deletion mutants that exhibited either impaired or wild-type growth under DTT, namely (*tR(UCU)M2 and tH(GUG)G1)*, both members of multi-copy families designated the MC group. In addition to the two viable single gene deletions (*tR(CCU)J* and *tL(GAG)G*), the initiator methionine *tiM(CAU)C* also demonstrated improved growth; we thus designated these three strains the SC group.

**Figure 5:**
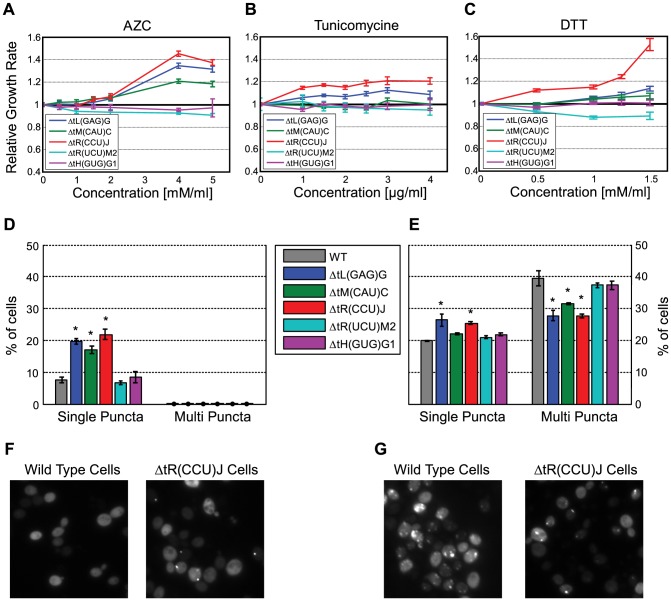
Changes in the tRNA pool affect protein folding. (A–C) Relative growth rate (compare to wild-type) of the following five deletion strain: *tL(GAG)G* (blue), *tR(CCU)J* (red), *tiM(CAU)C* (green), *tH(GUG)G1* (magenta) and *tR(UCU)M2* (cyan). Strains were grown in media supplemented with increasing concentrations of the following proteotoxic agent: AZC (A) Tunicamycin (B) DTT (C). (D) Percentage of cells that contain puncta in the populations of the above strains. (E) Percentage of cells that contain puncta in the populations of the above strains following treatment with 2.5 mM AZC. Data are presented as mean of 3 biological repetitions +/− SEM, in each repetition 500 cells were counted. (*) P<0.001 by Students *t*-test. (F–G) Images of representative fields for the wild-type and *tR(CCU)J* deletion strain, without treatment (F) and following treatment with 2.5 mM AZC (G).

The various strains were treated with either DTT, Azetidine 2 carboxylic acid (AZC)- a toxic analog of proline [44], or Tunicamycin- a drug used to induce the unfolded protein response (UPR) in the endoplasmic reticulum (ER) [45]. The growth of each strain was characterized under each proteotoxic agent applied at several concentrations. The strains in the MC group demonstrated either growth impairment or wild-type growth under all examined conditions. However, the deletions of single-copy tRNAs and to some extent the imitator methionine demonstrated reduced sensitivity to all three proteotoxic agents (Figure 5A–C). The differences in relative growth for all the examined strains were apparent even at low concentrations and were consistent upon increase in the concentrations of these proteotoxic agents (Figure 5A–C).

The fact that the tRNA deletion strains from the SC group are resistant to proteotoxic agents led us to hypothesize that deleting these genes might inflict intrinsic and chronic misfolding stress, even at the absence of the drug. This stress results in the activation of relevant cellular response that protects cells from the aggravating effect of extrinsic proteotoxic stress. Such an effect is reminiscent of the cross protection effect observed between environmental stressors [46], yet here it is manifested between a genetic perturbation and an environmental stress.

To directly examine whether changes in the tRNA pool induce proteotoxic stress in these strains, we examined the state of the protein quality control machinery using the naturally unstructured human protein VHL as a proteotoxic stress reporter [47]. In this system, the VHL protein can be destined to one of two cellular localizations. If the cell experiences protein-folding stress, the heterologous protein VHL will aggregate in inclusions (or puncta) due to saturation of the protein quality control machinery. In contrast, under normal conditions, the quality control machinery is available to properly deal with this naturally unfolded heterologous protein, thus it remains soluble in the cytoplasm and no inclusions are formed. For each of the five deletion strains, we quantified the number of VHL inclusions (puncta) in populations of yeast cells. This analysis revealed that indeed the tRNA deletions in the SC group exhibited a significant increase in the number puncta containing cells relative to the wild-type (Figure 5D and 5F), indicating saturation of the quality control machinery caused by intrinsic proteotoxic stress. The MC group did not exhibit inherent proteotoxic stress; their puncta containing cells count resembled that of the wild-type.

The inherent chronic proteotoxic stress observed for the SC deletions might provide them with the capacity to respond better to an additional external proteotoxic stress. To further explore this possibility we examined the state of the protein quality control machinery upon extrinsic proteotoxic stress induced by treatment with AZC. Treating the wild-type cells with AZC resulted in a rapid accumulation of the VHL protein in stress foci, indicated by increase in the occurrence of multiple inclusions [48]. As anticipated, the behavior of the SC group demonstrated a significant increase in the presence of a single punctum upon AZC treatment, however the appearance of stress foci (multi-puncta) was significantly lower compared to the wild-type and to the MC group (Figure 5E and 5G). As in the previous experiment, the deletions of the MC group responded in a similar manner to that of the wild-type, displaying increased number of stress foci.

These results thus indicate that the deletion of some tRNA genes induced an inherent proteotoxic stress in the cell, demonstrating a physiological role of proper tRNA supply in protein folding by an undetermined mechanism. Such physiological response renders these cells relatively less sensitive, compared to other tRNA deletion strains and the wild-type, from the otherwise harmful effect of proteotoxic drugs.

### Different molecular responses to deletions of tRNAs from single and multiple copy families

**Figure 6:**
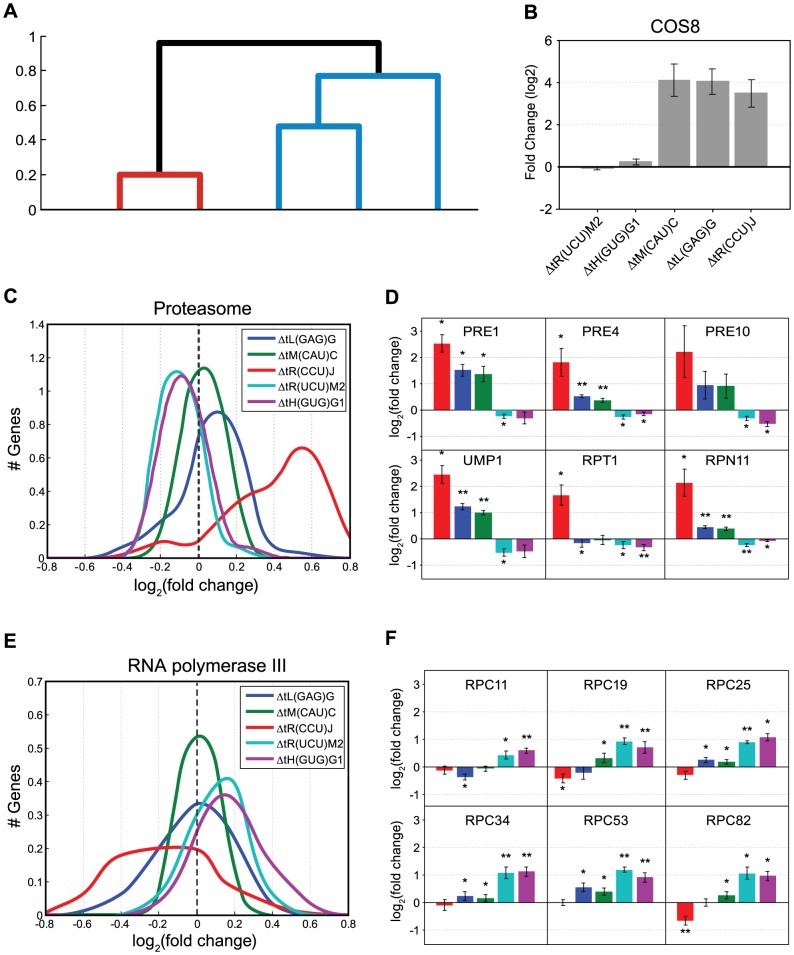
Molecular response to changes in the tRNA pool. (A) Dendrogram created by clustering changes in gene expression for five representative deletion strains, for more information see Materials and Methods. (B) Fold change of the *COS8* (*YHL048W*) mRNA levels in each of the five deletion strains as measured by microarrays. (C) The fold change distribution of mRNA levels as measured by microarrays, of genes composing the Proteasome pathway by the KEGG database [49], for each of the listed tRNA deletion strains. (D) mRNA Fold change of 6 representative genes from the proteasome pathway measured by RT-qPCR. Presented values are the mean of 3 biological repetitions +/− SEM. The strain colors are as in (C). If the mRNA fold change in a specific strain is significantly different from 0 (*t*-test) it is marked with:* (p<0.05) or ** (p<0.005). (E) The fold change distribution of mRNA levels as measured by microarrays, of genes composing the Pol III RNA Polymerase machinery module by the KEGG database, for each tRNA deletion strain. (F) mRNA Fold change of 6 representative genes from the Pol III KEGG module measured by RT-qPCR. Presented values are the mean of 3 biological repetitions +/− SEM. The strain colors are as in figure (C). If the mRNA fold change in a specific strain is significantly different from 0 (*t*-test) it is marked with:* (p<0.05) or ** (p<0.005). In all the sub-figures (C,D,E,F) values are plotted for the same five deletion strains: *tL(GAG)G* (blue), *tR(CCU)J* (red), *tiM(CAU)C* (green), *tH(GUG)G1* (magenta) and *tR(UCU)M2* (cyan).

To determine whether changes in the tRNA pool result in a distinct molecular signature, we examined the same set of tRNA deletions (SC and MC groups) using mRNA microarrays. For each strain, we measured genome-wide changes in mRNA levels compared to the wild-type, under rich growth conditions. The expression changes we observed were modest and demonstrated a correlation between the essentiality of the tRNA gene and the extent of changes in mRNA expression upon its loss. Hierarchical clustering of the strains according to similarity in expression changes (Fig. 6A and supplemental figure S8), revealed that the strains could be divided into two groups recapitulating the division to the SC and MC groups. An example for this division can be found in the pronounced effect observed for the *COS8* gene. This gene was extremely up-regulated (about 16 fold) in the SC group while unchanged in the MC group (Figure 6B). These results suggest different molecular signatures for the two groups, which are also related to the proteotoxic stress response.

To determine the responses and the underling molecular pathways that differentiate these two groups, we examined which KEGG pathways [49], [50] differentiate between them. We used Gene Set Enrichment Analysis (GSEA), a computational software which determines whether a defined set of genes shows statistically significant differences between two biological states [51], [52]. This analysis revealed a somewhat opposite signature between the two groups (Table 1 and supplemental figure S8). Pathways which are responsive to proteotoxic stress such as the Proteasome (FDR q-value<1E-5) and Protein processing in endoplasmic reticulum (FDR q-value 2E-3) were significantly induced in the SC group relative to the MC group. While in the MC groups, translation-related pathways such as Ribosome biogenesis (FDR q-value<1E-5) and Ribosome (FDR q-value 1E-4) were significantly induced compared to the SC group.

**Table 1:** KEGG pathways differentiating between tRNA deletion sets.

Higher in SC than in MC	Higher in MC than in SC
Proteasome (<1E-5)	Ribosome biogenesis in eukaryotes (<1E-5)
Oxidative phosphorylation (<1E-5)	RNA polymerase (<1E-5)
Endocytosis(2E-3)	Phenylalanine, tyrosine and tryptophan biosynthesis (<1E-5)
SNARE interactions in vesicular transport (2E-3)	Pyrimidine metabolism (5E-5)
Protein processing in endoplasmic reticulum (2E-3)	Ribosome (1E-4)
Starch and sucrose metabolism (2E-3)	Lysine biosynthesis (1E-4)
Citrate cycle (TCA cycle) (0.01)	Histidine metabolism (4E-4)
Meiosis (0.01)	Cysteine and methionine metabolism (4E-4)
Homologous recombination (0.02)	Riboflavin metabolism (5E-3)
Mismatch Repair (0.02)	Arginine and proline metabolism (8E-3)
Cell cycle (0.02)	Valine, leucine and isoleucine biosynthesis (0.01)
MAPK signaling pathway - yeast (0.02)	Purine metabolism (0.03)
Fructose and mannose metabolism (0.02)	Sulfur metabolism (0.03)
Nitrogen Metabolism (0.02)	Tyrosine Metabolism (0.03)
Phagosome (0.03)	Folate biosynthesis (0.04)

KEGG pathways [49] for which changes in genes expression are significantly different between the two groups of tRNA deletion strains: MC (multi-copy) group (*ΔtH(GUG)G1* and *ΔtR(UCU)M2*) vs. SC (single-copy) group (*ΔtL(GAG)G*, *ΔtR(CCU)J*, *ΔtiM(CAU)C*) calculated with GSEA [51], [52]. In the first column are pathways, which are higher in SC vs. MC and vice versa in the second column. The values are corrected for multiple hypothesis and the FDR q-values are indicated next to each pathway.

To further characterize these differences we focused on specific pathways. A more detailed examination of the expression changes observed for all the genes that constitute the proteasome complex revealed an up-regulation to various extents in response to deletion of tRNAs from the SC group. The MC group demonstrated no change and even a slight down-regulation of these genes (Figure 6C), a trend which was further verified using RT-qPCR (Figure 6D). These observations establish the notion that cells experience proteotoxic stress upon deletion of members of the SC group. A further indication of proteotoxic stress in these deletion strains is the up regulation of *COS8*. The exact biological function of this gene is still unclear, it was however found to interacts with *IRE1*, which is a hallmark regulator of the unfolding stress response [53].

An interesting distinction between the groups was also observed in the pathway consisting of the RNA polymerase machinery. Expression of genes that belong to this pathway were up-regulated only in the MC group (Table 1). Separating the RNA polymerase genes into modules corresponding to the different polymerases, revealed an interesting pattern. While the genes that encode RNA Pol II subunits did not change in any of the tRNA deletion strains (Supplemental figure S9), the genes encoding RNA Pol III machinery (the polymerase responsible for tRNA gene transcription) demonstrated up-regulation in the MC group and no change or even down regulation in the SC group (Figure 6E). These results were further verified by RT-qPCR (Figure 6F). Up-regulation of the pol III machinery for the MC group may suggest that in some MC deletion strains, the transcription of the remaining tRNA genes could increase, thus providing a possible molecular mechanism for backup compensation within families. Such response to deletions of tRNAs from the MC group could indicate the presence of a negative feedback loop, allowing the cell to respond to changes in the tRNA pool in the attempt to regain steady state levels.

## Discussion

In this study, we investigated the genetic architecture of the tRNA pool and its effect on cellular fitness using a comprehensive tRNA deletion library. We found extensive dispensability of many tRNA genes, especially under optimal growth conditions. Such lack of essentiality has been studied in protein-coding genes, and is often interpreted to reveal a role for partially redundant genes and pathways providing backup compensation for the deleted gene [33], [34], [54]–[56]. Similar design principles are displayed in the architecture of tRNA genes, which exhibited significant gene redundancy and compensation (either partial or complete) among family members. An additional reason for apparent lack of essentiality of genes is the limited set of examined environmental challenges, and it was indeed shown for protein-coding genes that challenging gene deletion libraries to less favorable conditions exposes more essentiality [57], [58]. We showed that a similar situation holds for tRNA genes. We found condition-specific functional roles for tRNAs, demonstrating increased demand for certain tRNA genes under certain defined conditions. This implies that the compensation within tRNA families changes across conditions. Such changes in the essentiality of tRNA genes can imply that the tRNA pool is dynamic and changes across conditions to accommodate cellular needs, as was recently suggested [59].

Further, we have discovered interesting architecture within families, which questions the prior notion that all tRNA gene copies contribute equally to the pool. Previous work has shown that Pol III transcription machinery displays different occupancy levels at various copies of the same tRNAs in the genome [21], [22], [60]. However, the potential phenotypic consequences of such transcriptional differences have not been previously explored. We report that the flanking sequences around each tRNA gene contains motifs that are predictive of the deletion phenotypic consequences, potentially affecting pol III transcription machinery.

We further speculate that some tRNA genes, i.e. the Major copies, might be active across all conditions and with only partial functional redundancy, thus their loss cannot be fully compensated. Minor copies on the other hand are either not transcribed or have a modest contribution to the tRNA pool, with complete functional redundancy by other copies, thus their loss can be fully compensated. Such architecture could provide the cell with means to respond in a dynamic manner to changes in the environment, by transcribing varying portions of the members of each tRNA family depending on demand. As such, differential contribution within tRNA families exposed an additional novel mean to regulate the tRNA pool and as a consequence to regulate the translation process.

An interesting finding was that changes in the tRNA pool elicit molecular changes in the cells even when no severe phenotype is detected. Our results demonstrated two distinct molecular signatures which can be attributed to the family architecture and the severity of the changes in the pool. Upon deletion of the two viable single copy tRNAs, and also upon deletion of one of the initiator tRNA methionine copies, the cell exhibited a response reminiscent of a proteotoxic stress. We were able to identify such a stress in these mutant cells. Although the exact mechanisms by which changes in the tRNA pool induces proteotoxic stress remains to be determined, we hypothesize that the elimination or reduction in these tRNAs may lead to events of amino acid misincorporation, ribosome frame-shifting or stalled protein synthesis terminations. Such events would have a clear impact on the protein quality control machinery of the cell by titrating chaperons to deal with misfolded or misassembled proteins. Translation errors such as incorrect tRNA selection and incorrect tRNA aminoacylation have been shown to induce proteotoxic stress in yeast [61], [62]. Given that cells exploit chaperon availability as a sensing mechanism to induce a stress response [63], [64], translation errors may lead to the onset such a response. On the other hand, deletions of tRNAs from multi-copy families results in milder effects on the tRNA pool due to the extensive redundancy or backup-compensation, and they indeed elicit a different cellular response from the one invoked upon deletion of single-member families. In the response to deletion of members from multi-gene families, the pol III transcription machinery seems to be up regulated. Such up-regulation would bring about induced transcription of tRNAs, this would act as a feedback mechanism to bring the tRNA pool closer to its normal state [65]. At least in one case (Supplemental figure S5) our results suggest the existence of such responsive backup among tRNA genes from the same family. Yet, a clearer relationship between changes in the tRNA pool, pol III activation, and tRNA transcription is still lacking. Regardless of the actual mechanism that determines the exact cellular response to tRNA deletions, the fact that such a response wiring exists may be beneficial for maintaining cellular robustness upon environmental changes and mutations.

This work provides for the first time a systemic tool to study the functional role of individual tRNA genes. Using this deletion library, we discovered a much more complex picture than was previously known. We anticipate that a high throughput mapping of all genetic interactions between pairs of tRNA genes (as done for protein-coding genes) [66], [67] would reveal the full genetic network. In addition, it might reexamine and potentially refine the wobble interaction rules from a genetic, rather than the traditional biochemical/structural perspective. The design principles defined in this study, consisting of massive gene redundancy as well as differential contribution of gene copies may provide cellular plasticity and allow the tRNA pool to accommodate various growth conditions and developmental planes. Deciphering the effects of tRNA variations as is found in some diseases such as cancer [68] and Huntington [69] can provide possible routes for future treatment. We provide this novel set of minimalist genetic perturbations in the translation machinery as a resource to the yeast community towards further characterization of this highly complex process as well as additional cellular processes.

## Materials and Methods

### Creation of tRNA deletion library

The complete tRNA pool of *S. cerevisiae* was obtained from the tRNA genomic database [70], where 286 tRNA genes are annotated. 13 tRNA genes are encoded by the mitochondrial genome and the remaining are nuclear-encoded. Here we focused on the nuclear-encoded tRNAs. Two tRNA genes that are annotated in this database as not determined, belong to the *tS(GCU)* family. Thus, the *tS(GCU)* family contains two additional members, *tS(GCU)L* and *tS(GCU)D* , both verified by PCR, bringing the total number of nuclear encoded tRNA genes to 275. Deletion strains were constructed using a PCR-based gene deletion [71], [72], in the genetic background of the Y5565 strain (*MATα, can1Δ::MFA1pr-HIS3, mfα1Δ::MFα1pr-LEU2, lyp1Δ, ura3Δ0, leu2Δ0*). The *S. cerevisiae* strain S288C reference genome sequence R57-1-1 downloaded from the Saccharomyces Genome Database was used for primer design. Each deletion construct contained 45 bp flanking or overlapping a tRNA sequence for specific recombination event, a unique barcode and the HPH antibiotics ‘cassette’, conferring resistance to the antibiotic hygromycin B, [73]. PCR products were transformed into yeast cells and single colonies were verified by PCR. Three colonies from each strain were used to verify phenotypes in growth analysis. A wild-type strain in which the same antibiotic marker was integrated 200 bp upstream of the *tL(CAA)L3* locus was created as a control and was used in all analyses as wild-type. A complete list of all plasmids, yeast strains and PCR fragments can be found in Supplemental text S1 and Supplemental table S5.

### Measurements of growth using OD reads

Strains were grown for two days at 30°C in YPD (1% yeast extract, 2% peptone, 2% glucose), diluted (1∶50) into the appropriate medium in U-bottom 96-well plates and grown at 30°C (using TECAN Freedom EVO robot). The OD of the population in each plate was monitored every 30 minutes using a spectrophotometer at 600 nm (INFINITE200-TECAN). Each plate contained a wild-type strain to which the growth parameters of the deletions strains were normalized. The OD reads served for growth analysis and extraction of growth parameters. At least 3 biological repeats and 36 technical repeats were performed for each strain in each condition. Complete description of analysis and normalization procedures are provided in the Supplemental text S1.

### Yeast growth conditions

Library strains were screened in the following growth conditions: YPD, SCD (0.67% Bacto-yeast nitrogen base w/o amino acids 2% glucose supplemented with amono acids), YP supplemented with 0.025% glucose, YP supplemented with 1% galactose, YPD supplemented with 0.5 M NaCl, SCD supplemented with 1.5 mM DTT. Growth measurements were also performed on YPD supplemented with increasing concentrations of the proteotoxic agents DTT, AZC and Tunicamycin.

### Motif analysis

A sequence motif analysis was performed using the MEME online software [43]. The motif search was done on the upstream sequence of tRNA genes which exhibited a yield impairment phenotype in rich medium upon deletion (42 genes) versus the upstream sequence of tRNA genes which exhibited a phenotype in no more than two out of the six conditions (99 genes). To apply location constrains on the motifs, the MEME analysis was done in windows of size 9 bp, looking for motifs of 4–8 bp in length.

### Analysis of protein quality control using VHL-CHFP reporter

Wild-type and tRNA deletion strains harboring the pGAL-VHL-mCherry (CHFP) fusion were grown overnight on SCD+2% raffinose, diluted into SCD+2% galactose and grown at 30°C for 6 hours. Cells were visualized using an Olympus IX71 microscope controlled by Delta Vision SoftWoRx 3.5.1 software, with ×60 oil lens. Images were captured by a Photometrics Coolsnap HQ camera with excitation at 555/28 nm and emission at 617/73 nm (mCherry). Images were scored using the ImageJan Image Processing and Analysis software. The percentage of cells harboring VHL-CHFP foci was determined by counting at least 500 cells for each strain in three biological repetitions. Protein un-folding stress was induced with AZC at a concentration of 2.5 mM AZC (Sigma) following induction with galactose.

### Analysis of genome wide expression changes

Cultures were grown in YPD medium at 30°C to a cell concentration of 1.5*10^7^ cells/ml. Cells were then harvested, frozen in liquid nitrogen, and RNA was extracted using MasterPure™ (EPICENTER Biotechnologies). The quality of the RNA was assessed using the BIOANALYZER 2100 platform (AGILENT); samples were then processed and hybridized to Affymetrix yeast 2.0 microarrays using the Affymetrix GeneChip system according to manufacturer's instructions. The background adjustment was done using the Robust Multi-array Average (RMA) procedure followed by quintile normalization.

For each strain, the fold change in expression for all genes was calculated by comparing the wild-type measurement in the same batch and averaged over two biological repeats.

### Microarray analysis

The cluster tree is based on the correlation between the mRNA fold change of the different strains. For the clustering we used the top 50% of the sorted genes based by the gene variance across the strains.

### Microarray data access

The data from this study have been submitted to the NCBI Gene Expression Omnibus (GEO) under accession number GSE47050. A list of the measured fold changes for all genes in each strain can be found in Supplemental table S4.

### RT-qPCR measurements

Cultures were grown in YPD medium at 30°C to a cell concentration of 1*10^7^ cells/ml. RNA was extracted using MasterPure™ (EPICENTER Biotechnologies), and used as a template for quantitative RT–PCR using light cycler 480 SYBR I master (Roche)(LightCycler 480 system) according to the manufacture instructions. A list of the primers can be found in Supplemental table S6.

## Supporting Information

Figure S1 Growth measurements parameters. (A) Schematic growth curve of Optical Density (OD) vs. time. The red dots indicate the time points from which the growth rate (1) and growth yield (2) parameters are extracted. (B) Dot plot for all strains in the library grown in YPD. Each strain is represented by a blue dot, showing its sigma growth rate vs. its sigma growth yield values. The Pearson correlation coefficient is −0.019 indicating there is no correlation between the two parameters p-val 0.794.
(PDF)

Figure S2 Phenotypes cannot be explained by family size and amino-acid identity. Sigma growth parameters for the tRNA library grown in rich medium are plotted in boxes sorted by either family size or amino-acid identity. For each box, the central mark is the median, the edges of the box are the 25th and 75th percentiles. Sigma growth yield by family size (A) sigma growth rate by family size (B) sigma growth yield by amino-acid (C) sigma growth rate by amino-acid (D). Apart from the singletons whose deletion strains are often lethal or impaired, we could not explain the observed growth phenotypes, in either growth rate or yield, by either the size of the family, or the amino acid identity.
(PDF)

Figure S3 tRNA deletion phenotype are not correlated to the expression of nearby genes. (A–B) the average expression level of the genes located upstream and downstream to the tRNA gene that was deleted in each strain vs. the sigma growth yield (A) or the sigma growth rate (B). (C) Relative growth parameters of *tR(CCU)J* deletion (black), *tR(CCU)J* deletion containing a centromeric plasmid harboring the *tR(CCU)J* gene (gray) and a strain deleted for the *Y* *JR055W* gene which is the protein-coding gene located downstream of *tR(CCU)J* (white). As can be seen only the *tR(CCU)J* deletion strain exhibits growth rate impairment while the two other strains do not.
(PDF)

Figure S4 Single tRNA genes can sustain wild-type growth upon deletion of multiple members in three gene families. (A–B) Relative growth rate (red) and growth yield (blue) values of double deletion combinations containing members of the *tG(UCC)* family (A) and the *tS(UGA)* family (B). In each experiment the mean of 3 biological repetitions is presented +/− SEM. Two σ around the mean of the wild-type are indicated by red and blue lines around 1 (wild-type value).
(PDF)

Figure S5 Compensation within some tRNA families is due to plasticity of the pool and transcriptional changes of the remaining copies. RT-qPCR measurement of the RNA levels of the *tS(UGA)* family(A) and *tL(UAG)* family (B) upon deletion of various members of the family. Results are reported in terms of log2 fold change of the expression level in each of the indicated deletion strain compared to the wild-type. In both (A) and(B) the * indicates cases in which the fold change was significantly different from zero (*t*-test, p-value<0.05).
(PDF)

Figure S6 Epistasis of trm9 deletion with Glutamic Acid tRNAs. Examining a more general role for Trm9 in modulating the compensation between tRNA families we chose the second tRNA family that is modified by Trm9, *tE(UUC)*, and in addition we examined the *tE(UCU)* family. Together these two families decode in a split codon box, in a similar manner to the Arginine UCU and CCU families. We created 10 double deletions, each consisting of the enzyme along with one of the tRNA genes of the two glutamic acid families and analyzed their interactions by epistasis. Epistasis values for co-deletion strains which contain the deletion of trm9 with: the deletion of the two members of *tE(CUC)* family, and eight members of the *tE(UUC)* family. Epistasis values of the relative growth yield and growth rate are indicated in grey and green respectively. Data is presented as mean +/− SEM of 3 independent experiments.
(PDF)

Figure S7 Identical tRNA genes contribute differentially to the tRNA pool. (A–B) Growth rate values of the tRNA deletion library in rich medium (A) and low glucose (B) sorted by families and amino-acid identity. The horizontal lines denote two standard deviations around the mean of the wild-type in that condition. Dots above or below these lines are considered phenotypes. (C) Relative growth yield values (data of 3 biological repetitions +/− SEM is presented) of five *tR(UCU)* deletion strains (Grey) and the corresponding complementation strains (White). Each complementation strain carries the deleted tRNA gene on a centromeric plasmid. The values are relative to the wild-type. In the complementation experiment, the wild-type harbors an empty plasmid. (D) Relative growth yield values of strain deleted for *tR(UCU)M2* gene (a major copy of the *tR(UCU)* family- marked as *ΔM2*), and *ΔM2* strains containing different centromeric plasmids. Each centromeric plasmid carries the *tR(UCU)* tRNA flanked from each side by 200 bp sequence identical to a the different members of the *tR(UCU)* family.
(PDF)

Figure S8 Expression changes of tRNA deletions. Expression changes for the five deletion strains. Each row indicates a gene and each column is a tRNA deletion strain. The genes and strains are sorted according to the clustering results (see Materials and Methods). The Color bar indicates the log2 fold change. The groups of genes enriched for relative pathways are indicated on the right (locations were found by looking at the highest hypergeometric enrichments for varying window sizes).
(PDF)

Figure S9 Fold change of the Pol II pathway. (A) The fold change distribution of mRNA levels as measured by microarrays, of genes composing the Pol II RNA Polymerase machinery by the KEGG database for each of the listed tRNA deletion strains. (B) mRNA Fold change of 3 representative genes from the Pol II pathway measured by RT-qPCR. Presented values are the mean of 3 biological repetitions +/− SEM. The strain colors are as in figure (A). If the mRNA fold change in a specific strain is significantly different from 0 (t-test) it is marked with:* (p<0.05) or ** (p<0.005). In both sub-figures (A, B) values are plotted for the same five deletion strains: *tL(GAG)G* (blue), *tR(CCU)J* (red), *tiM(CAU)C* (green), *tH(GUG)G1* (magenta) and *tR(UCU)M2* (cyan).
(PDF)

Table S1 Correlation between tRNA phenotype and expression of nearby genes.
(DOC)

Table S2 List of all tRNA deletion strains in the library and their respective phenotypes across conditions.
(XLS)

Table S3 List of double deletion strains and their phenotypes.
(XLS)

Table S4 Microarray Fold change measurements for selected tRNA deletion strains.
(XLS)

Table S5 List of primers used to create the tRNA deletion strains.
(XLS)

Table S6 List of primers used for RT-qPCR experiments.
(XLSX)

Text S1 Supplementary methods and note.
(DOC)
