# Supplementary figures and images for: A Comprehensive tRNA Deletion Library Unravels the Genetic Architecture of the tRNA Pool
Source: PLoS Genet. 2014 Jan 16;10(1):e1004084. doi: 10.1371/journal.pgen.1004084 (PMC3894157; doi:10.1371/journal.pgen.1004084)

A

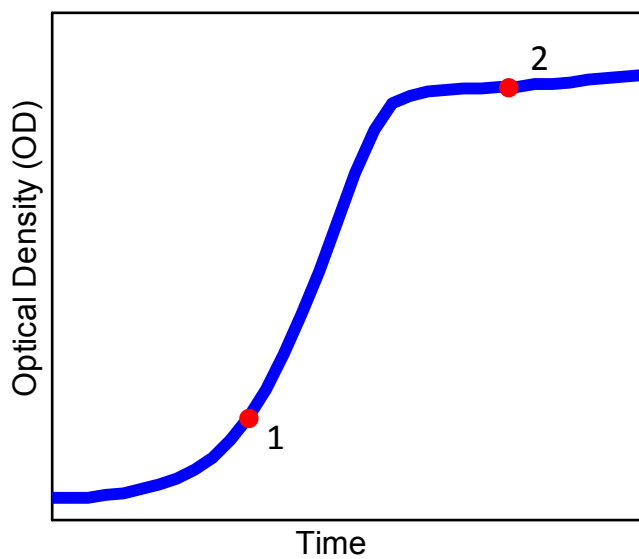

B

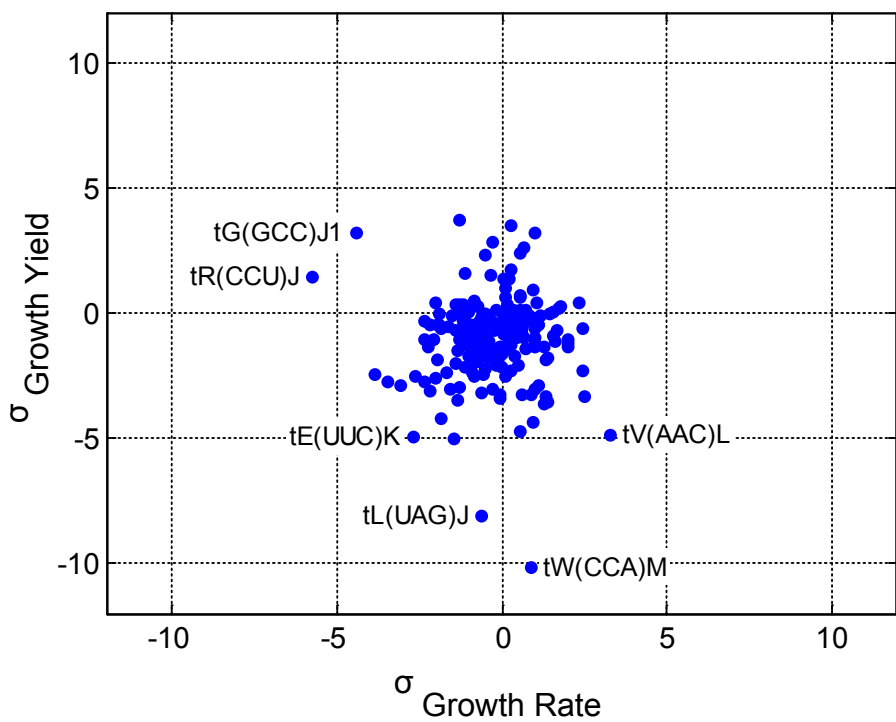

Supplement: Figure S1 — Growth measurements parameters. (A) Schematic growth curve of Optical Density (OD) vs. time. The red dots indicate the time points from which the growth rate (1) and growth yield (2) parameters are extracted. (B) Dot plot for all strains in the library grown in YPD. Each strain is represented by a blue dot, showing its sigma growth rate vs. its sigma growth yield values. The Pearson correlation coefficient is −0.019 indicating there is no correlation between the two parameters p-val 0.794. (PDF) [file pgen.1004084.s001.pdf]

A

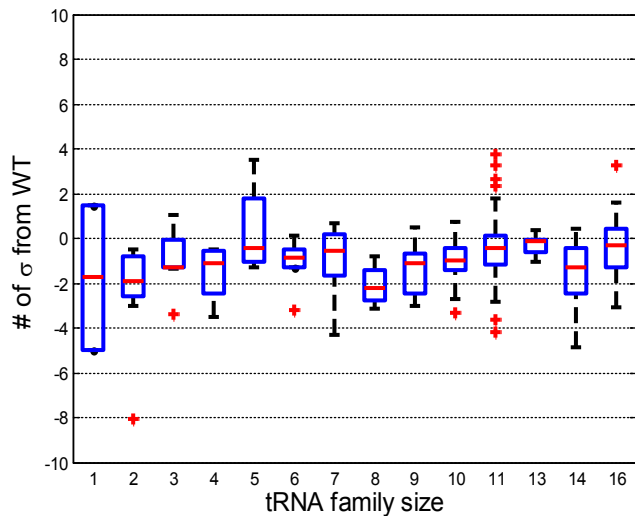

B

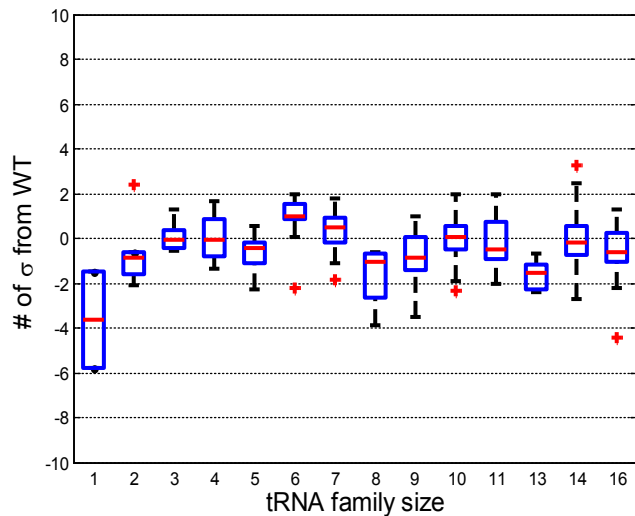

C

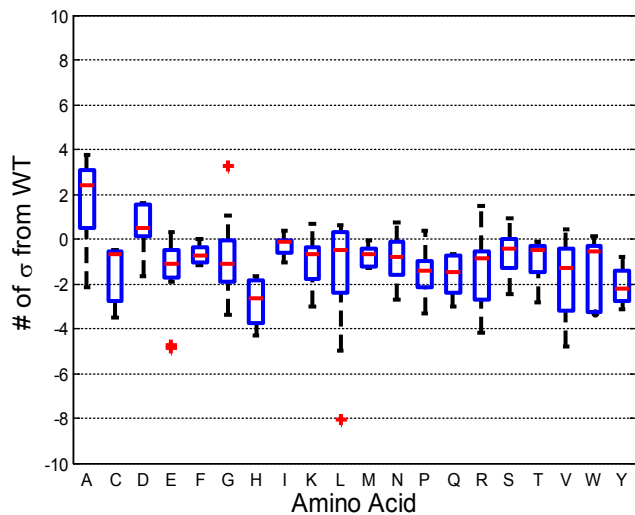

D

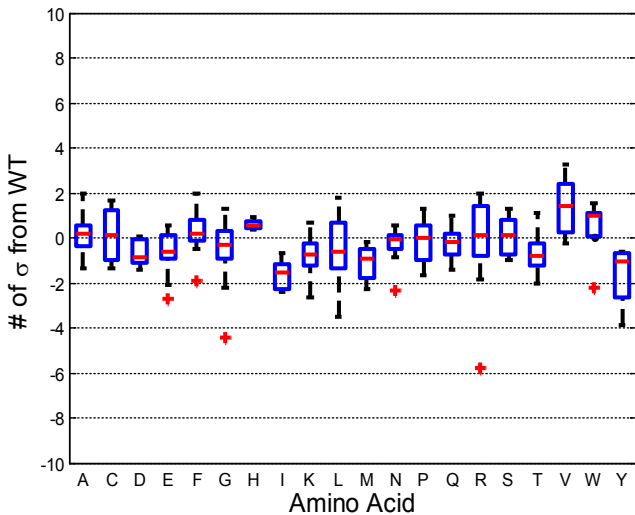

Supplement: Figure S2 — Phenotypes cannot be explained by family size and amino-acid identity. Sigma growth parameters for the tRNA library grown in rich medium are plotted in boxes sorted by either family size or amino-acid identity. For each box, the central mark is the median, the edges of the box are the 25th and 75th percentiles. Sigma growth yield by family size (A) sigma growth rate by family size (B) sigma growth yield by amino-acid (C) sigma growth rate by amino-acid (D). Apart from the singletons whose deletion strains are often lethal or impaired, we could not explain the observed growth phenotypes, in either growth rate or yield, by either the size of the family, or the amino acid identity. (PDF) [file pgen.1004084.s002.pdf]

A

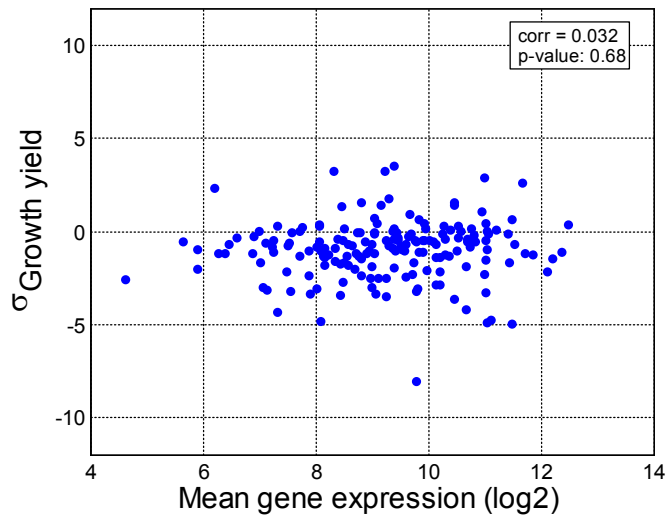

B

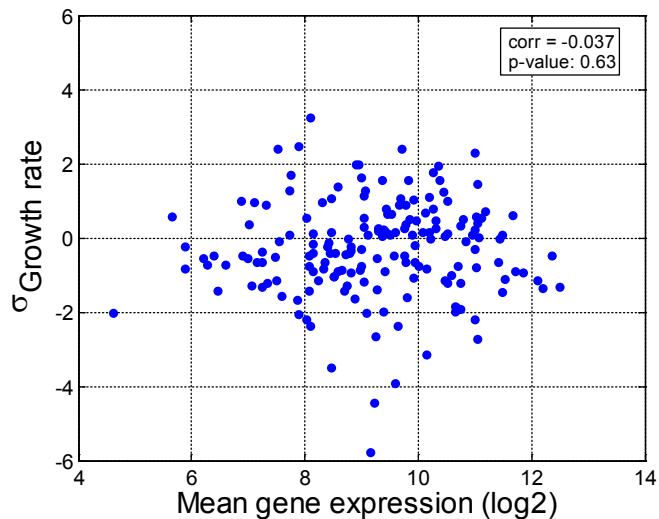

C

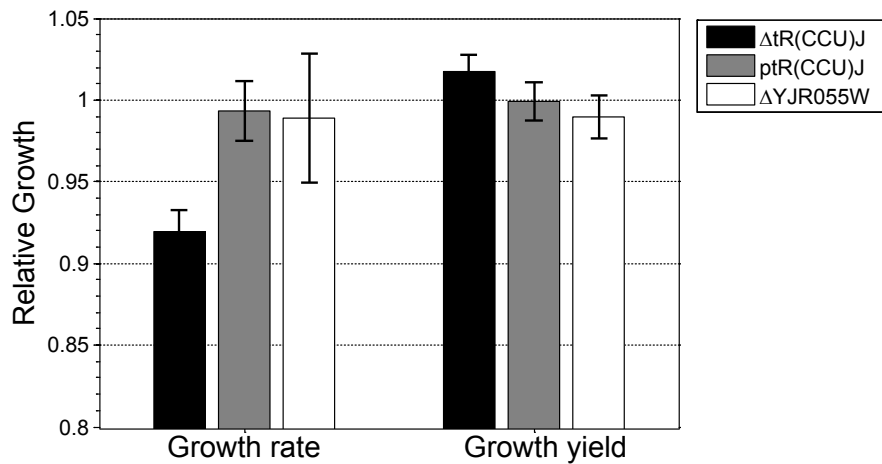

Supplement: Figure S3 — tRNA deletion phenotype are not correlated to the expression of nearby genes. (A–B) the average expression level of the genes located upstream and downstream to the tRNA gene that was deleted in each strain vs. the sigma growth yield (A) or the sigma growth rate (B). (C) Relative growth parameters of tR(CCU)J deletion (black), tR(CCU)J deletion containing a centromeric plasmid harboring the tR(CCU)J gene (gray) and a strain deleted for the Y JR055W gene which is the protein-coding gene located downstream of tR(CCU)J (white). As can be seen only the tR(CCU)J deletion strain exhibits growth rate impairment while the two other strains do not. (PDF) [file pgen.1004084.s003.pdf]

A

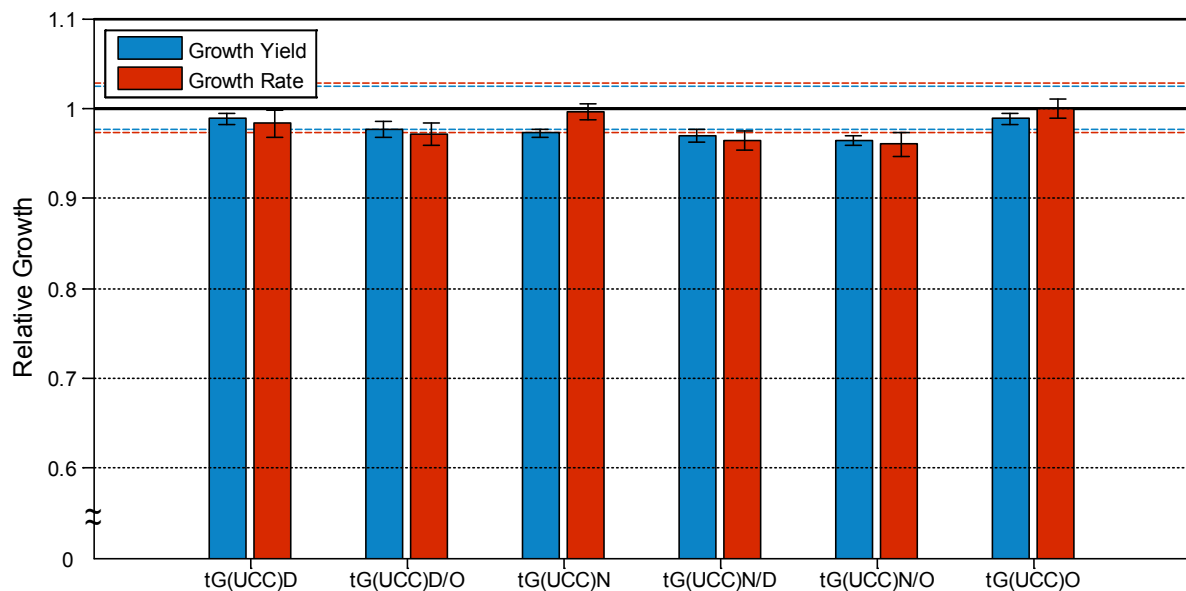

B

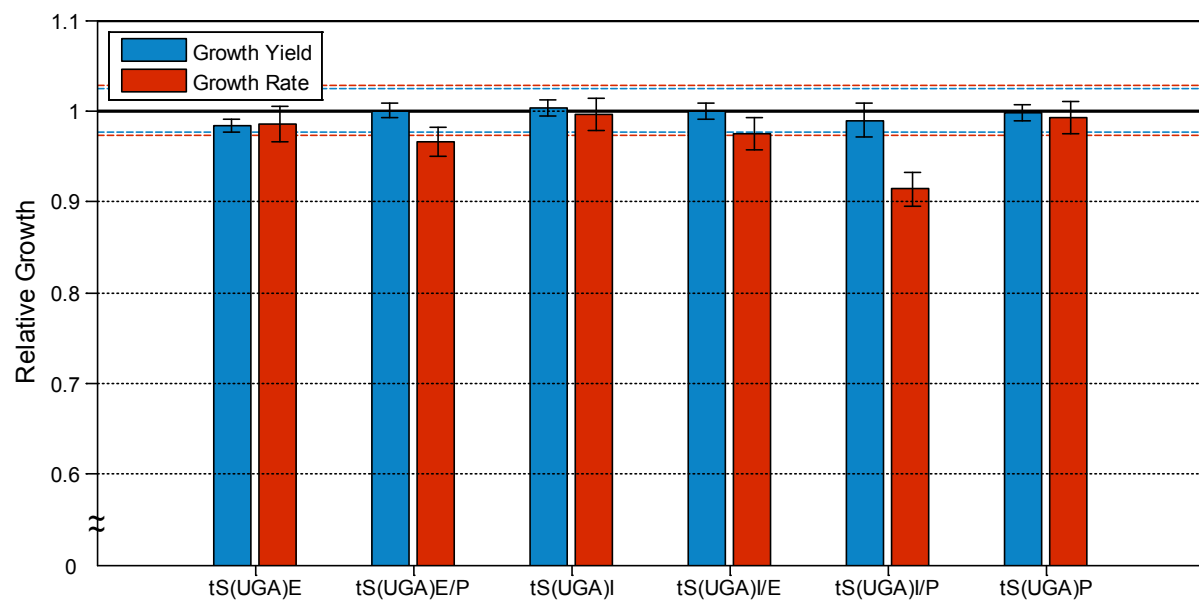

Supplement: Figure S4 — Single tRNA genes can sustain wild-type growth upon deletion of multiple members in three gene families. (A–B) Relative growth rate (red) and growth yield (blue) values of double deletion combinations containing members of the tG(UCC) family (A) and the tS(UGA) family (B). In each experiment the mean of 3 biological repetitions is presented +/− SEM. Two σ around the mean of the wild-type are indicated by red and blue lines around 1 (wild-type value). (PDF) [file pgen.1004084.s004.pdf]

**A** $\log_2(\text{fold change})$ 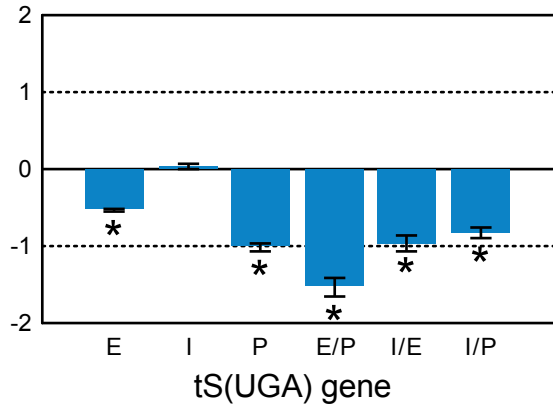**B** $\log_2(\text{fold change})$ 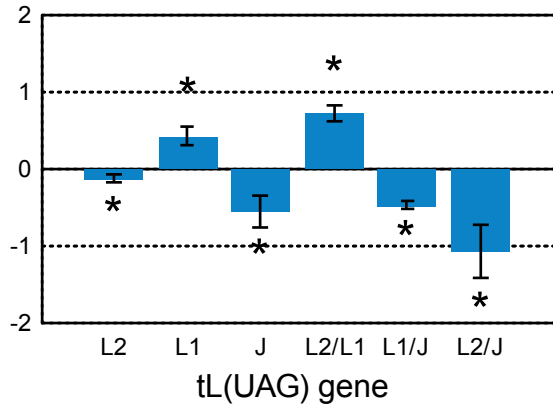

Supplement: Figure S5 — Compensation within some tRNA families is due to plasticity of the pool and transcriptional changes of the remaining copies. RT-qPCR measurement of the RNA levels of the tS(UGA) family(A) and tL(UAG) family (B) upon deletion of various members of the family. Results are reported in terms of log2 fold change of the expression level in each of the indicated deletion strain compared to the wild-type. In both (A) and(B) the * indicates cases in which the fold change was significantly different from zero (t-test, p-value<0.05). (PDF) [file pgen.1004084.s005.pdf]

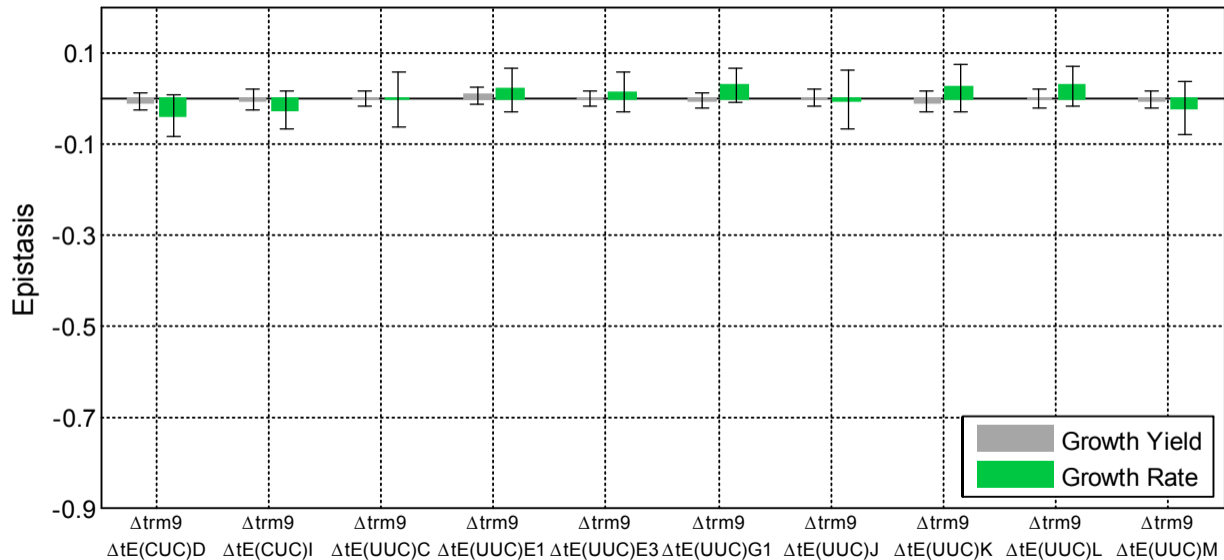

Supplement: Figure S6 — Epistasis of trm9 deletion with Glutamic Acid tRNAs. Examining a more general role for Trm9 in modulating the compensation between tRNA families we chose the second tRNA family that is modified by Trm9, tE(UUC), and in addition we examined the tE(UCU) family. Together these two families decode in a split codon box, in a similar manner to the Arginine UCU and CCU families. We created 10 double deletions, each consisting of the enzyme along with one of the tRNA genes of the two glutamic acid families and analyzed their interactions by epistasis. Epistasis values for co-deletion strains which contain the deletion of trm9 with: the deletion of the two members of tE(CUC) family, and eight members of the tE(UUC) family. Epistasis values of the relative growth yield and growth rate are indicated in grey and green respectively. Data is presented as mean +/− SEM of 3 independent experiments. (PDF) [file pgen.1004084.s006.pdf]

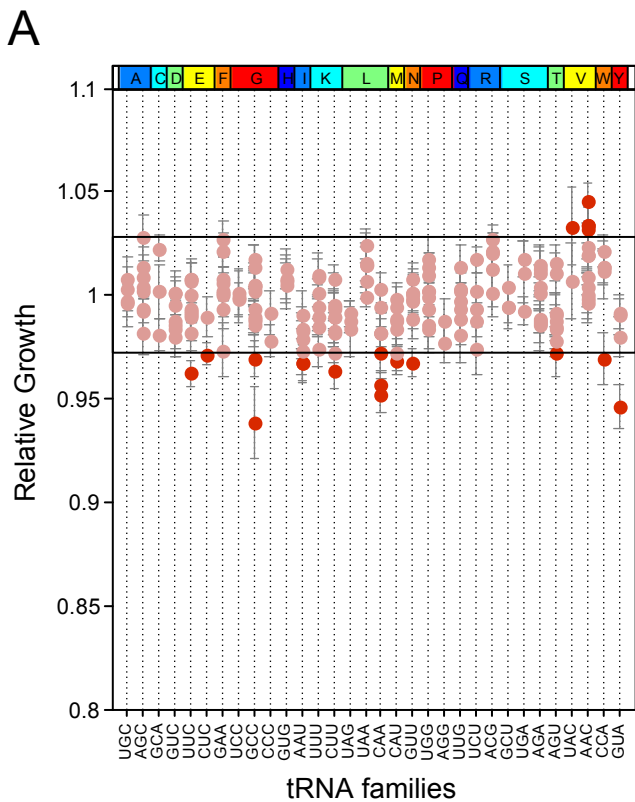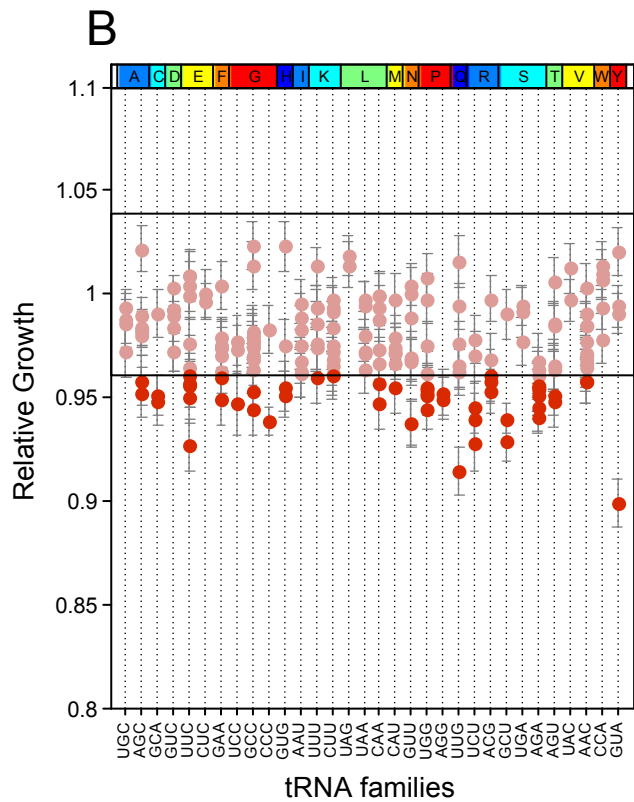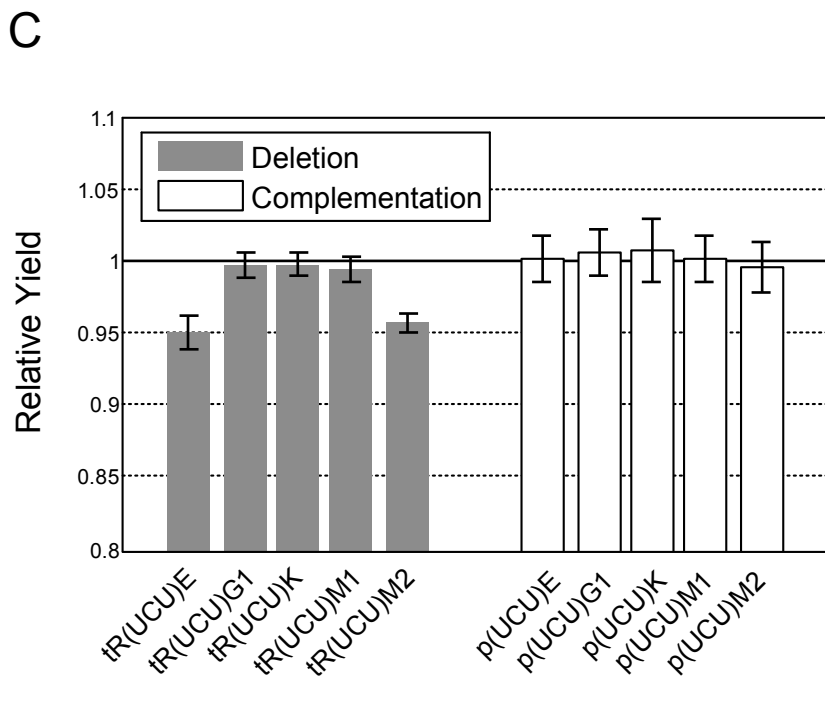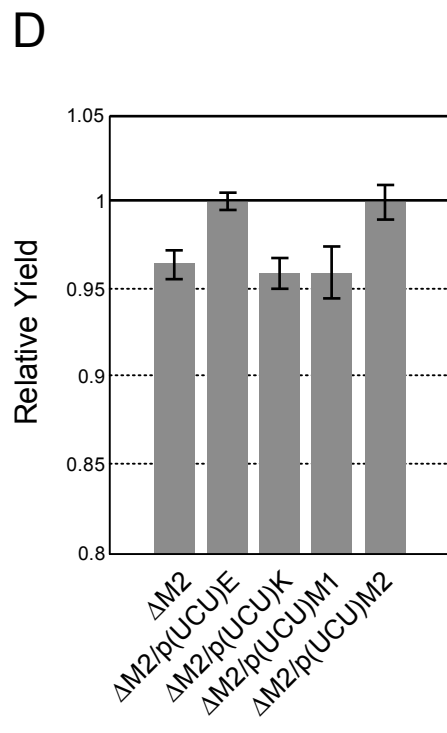

Supplement: Figure S7 — Identical tRNA genes contribute differentially to the tRNA pool. (A–B) Growth rate values of the tRNA deletion library in rich medium (A) and low glucose (B) sorted by families and amino-acid identity. The horizontal lines denote two standard deviations around the mean of the wild-type in that condition. Dots above or below these lines are considered phenotypes. (C) Relative growth yield values (data of 3 biological repetitions +/− SEM is presented) of five tR(UCU) deletion strains (Grey) and the corresponding complementation strains (White). Each complementation strain carries the deleted tRNA gene on a centromeric plasmid. The values are relative to the wild-type. In the complementation experiment, the wild-type harbors an empty plasmid. (D) Relative growth yield values of strain deleted for tR(UCU)M2 gene (a major copy of the tR(UCU) family- marked as ΔM2), and ΔM2 strains containing different centromeric plasmids. Each centromeric plasmid carries the tR(UCU) tRNA flanked from each side by 200 bp sequence identical to a the different members of the tR(UCU) family. (PDF) [file pgen.1004084.s007.pdf]

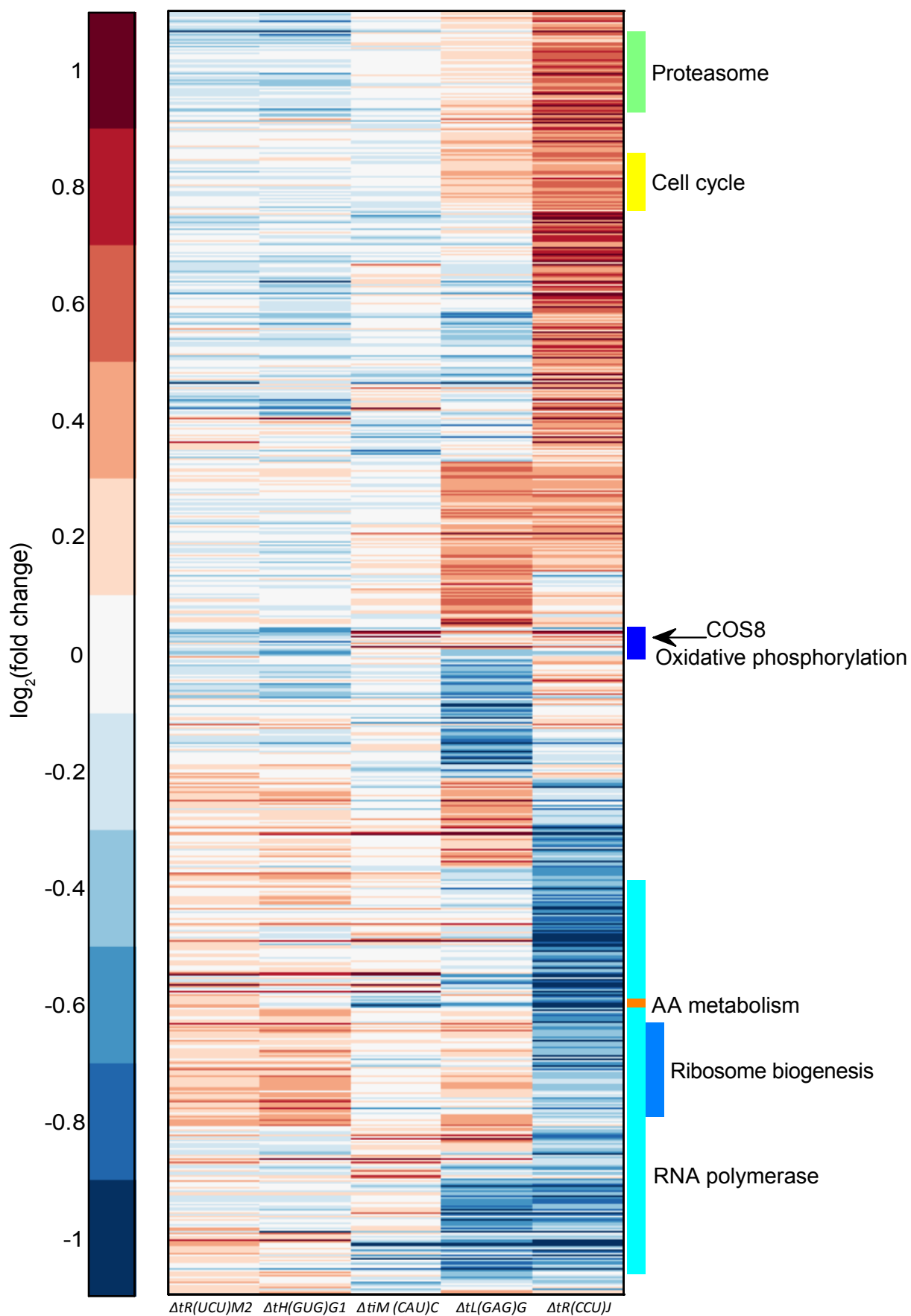

Supplement: Figure S8 — Expression changes of tRNA deletions. Expression changes for the five deletion strains. Each row indicates a gene and each column is a tRNA deletion strain. The genes and strains are sorted according to the clustering results (see Materials and Methods). The Color bar indicates the log2 fold change. The groups of genes enriched for relative pathways are indicated on the right (locations were found by looking at the highest hypergeometric enrichments for varying window sizes). (PDF) [file pgen.1004084.s008.pdf]

**A****RNA polymerase II**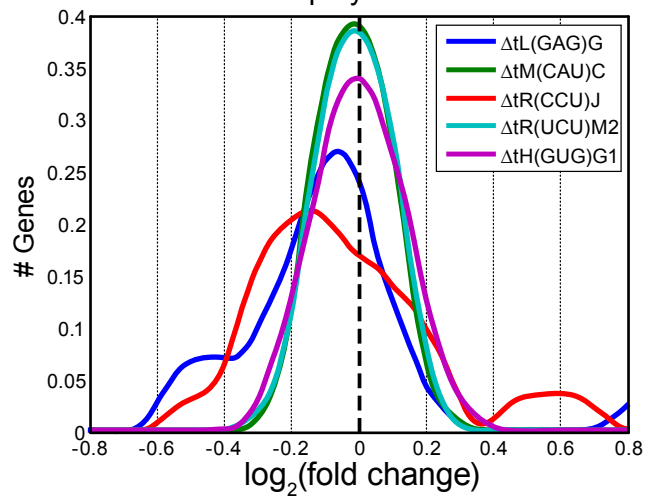**B****RNA Polymerase II****RPB2**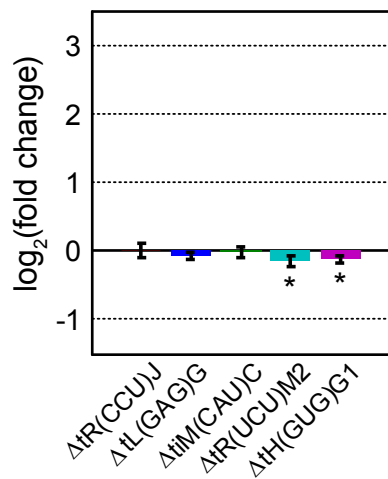**RPB3**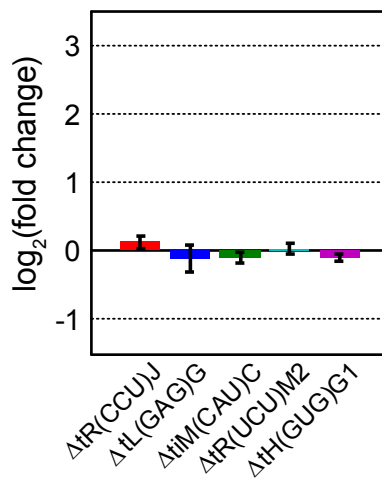**RPB9**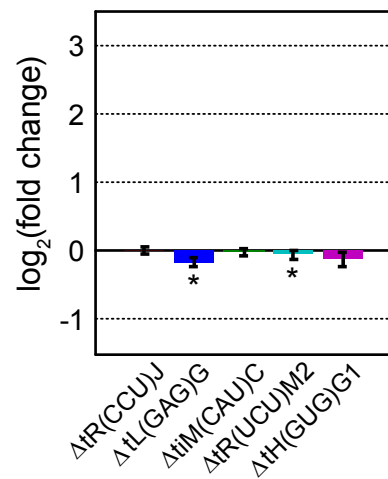

Supplement: Figure S9 — Fold change of the Pol II pathway. (A) The fold change distribution of mRNA levels as measured by microarrays, of genes composing the Pol II RNA Polymerase machinery by the KEGG database for each of the listed tRNA deletion strains. (B) mRNA Fold change of 3 representative genes from the Pol II pathway measured by RT-qPCR. Presented values are the mean of 3 biological repetitions +/− SEM. The strain colors are as in figure (A). If the mRNA fold change in a specific strain is significantly different from 0 (t-test) it is marked with:* (p<0.05) or ** (p<0.005). In both sub-figures (A, B) values are plotted for the same five deletion strains: tL(GAG)G (blue), tR(CCU)J (red), tiM(CAU)C (green), tH(GUG)G1 (magenta) and tR(UCU)M2 (cyan). (PDF) [file pgen.1004084.s009.pdf]
