# Supplementary material for: A Comprehensive tRNA Deletion Library Unravels the Genetic Architecture of the tRNA Pool
Source: PLoS Genet. 2014 Jan 16;10(1):e1004084. doi: 10.1371/journal.pgen.1004084 (PMC3894157; doi:10.1371/journal.pgen.1004084)
Supplement: Table S1 — Correlation between tRNA phenotype and expression of nearby genes. (DOC) [file pgen.1004084.s010.doc]

|  | **Upstream Gene** | **Downstream Gene** | **Average**  **(up & down genes)** | **Minimal distance** |
| --- | --- | --- | --- | --- |
| **yield** phenotype | r=0.06  p-value:0.41 | r=-0.02  p-value:0.78 | r=0.03  p-value:0.68 | r=0.06  p-value:0.36 |
| **absolute yield** phenotype (no matter whether it is impairment or improvement) | r=-0.03  p-value:0.71 | r=0.02  p-value:0.80 | r=0.01  p-value:0.95 | r=0.07  p-value:0.37 |
| **growth** phenotype | r=-0.01  p-value:0.92 | r=-0.05  p-value:0.50 | r=-0.04  p-value:0.63 | r=0.04  p-value:0.61 |
| **absolute growth** phenotype (no matter whether it is impairment or improvement) | r=-0.02  p-value:0.78 | r=-0.04  p-value:0.60 | r=-0.04  p-value:0.61 | r=0.12  p-value:0.08 |
| **any absolute** phenotype  (growth rate/growth yield impairment/ improvement) | r=-0.02  p-value:0.77 | r=-0.01  p-value:0.86 | r=0.00  p-value:0.99 | r=0.08  p-value:0.24 |

**Table S1. Correlation between tRNA phenotype and expression of nearby genes.**

The table shows the correlation (r) and the p-value between the deletion strain phenotypes and the expression of nearby genes. Each row shows the correlation between the sigma value of the growth yield/growth rate in rich medium (YPD) and the expression of the nearby gene/s taken from the microarray wild-type measurements (columns 1-3) and the distance to the closest genomic feature (column 4).
