# Supplementary material for: A Comprehensive tRNA Deletion Library Unravels the Genetic Architecture of the tRNA Pool
Source: PLoS Genet. 2014 Jan 16;10(1):e1004084. doi: 10.1371/journal.pgen.1004084 (PMC3894157; doi:10.1371/journal.pgen.1004084)
Supplement: Text S1 — Supplementary methods and note. (DOC) [file pgen.1004084.s016.doc]

# Supplemental Text

# Materials and Methods

**Yeast strains and plasmids**

- *S. cerevisiae* strain Y5565 *(MATα can1∆::MFA1pr-HIS3 mfα1∆::MFα1pr-LEU2 lyp1∆ ura3∆0 leu2∆0)* was used as the genetic background for the tRNA deletion library strains and the double deletion strains.
- *S. cerevisiae* strain BY4743 *(MATa/α his3Δ1/his3Δ1 leu2Δ0/leu2Δ0 LYS2/lys2Δ0 met15Δ0/MET15 ura3Δ0/ura3Δ0)* was used for analysis of tRNA essentiality in tetrad analysis.
- pAG32 DEL-MARKER-SET (EOUROSCARF) [1] was used to amplify the Hygromycin B resistance marker.
- pAG25 DEL-MARKER-SET (EOUROSCARF) [1] was used to amplify the Nourseothricin resistance marker.
- pFA6a-kanMX6 [2] was used to amplify the Kanamycin resistance marker.
- pRS316- Centromeric plasmid [3] was used for tRNA complementation assays.

**Creation of a tRNA deletion library**

In similarity to the yeast gene deletion library [8], the tRNA deletion library was constructed using homologous recombination, replacing each tRNA gene with the HPH antibiotics cassette. The DNA template for the recombination event was created using two sequential PCR reactions. The first PCR reaction created the basic template for the recombination containing the antibiotic marker and a 23bp sequence homologous to the desired tRNA. The second PCR reaction served to lengthen this template so it contains 45bp homologues to the desired tRNA deletion. The first PCR reaction used primers containing a genomic sequence that flanks either the 5' or 3' end of the tRNA (directly proximal and distal to the start and end of the gene respectively), 18 and 17bp of sequence common to all gene disruptions as was used in the yeast deletion collection, a 20 base pairs unique sequence (the 'molecular bar-code' TAG) and 22 base pairs of sequence, homologous to the HPH gene. The template for this PCR reaction was the pAG32 plasmid, encoding the hygromycin B phosphotransferase conferring resistance to the antibiotic hygromycin B. The second PCR reaction used two tRNA gene-specific 45 bp primers, to extend the tRNA specific homology of the first PCR product to a total of 45 bp. The product of the second PCR served for yeast transformation. For a full list of primers see Supplemental table S5.

**Verification of tRNA deletion strains**

For each tRNA deletion strain, five different colonies were selected, and verified by two PCR reactions. The junctions of the antibiotics cassette were amplified using two primer combinations. Each primer combination contained a primer homologous to sequence within antibiotics cassette of either the promoter or terminator of the marker gene, and a tRNA specific primer, homologous to sequence upstream or downstream of the tRNA deletion. Only colonies from which the correct products were amplified served for further analysis.

**Verification of lethality of tRNA deletions by tetrad analysis**

To confirm the essentiality of double and triple tRNA deletions, diploid strains were created by mating two deletion strains, either single or double deletions. These heterozygous diploids were sporulated in SC medium (1% potassium acetate) for three days at 25°C. The resulting tetrads were then dissected using a Micromanipulator (Singer). Spores were then allowed to germinate at 30°C on YPD plates and scored on plates containing the appropriate antibiotics. The essentiality of single tRNA genes was confirmed by deletion of the gene from a diploid strain, BY4347, in a similar manner to the construction of the tRNA deletion library. The resulting colonies were verified, grown on rich medium, sporulated and scored. At least 100 tetrads were scored for each such deletion.

## Construction of complementation plasmids

In order to reintroduce into the cell a tRNA deleted gene we created a set of “complementation plasmids”. Complementation plasmids were constructed for multiple tRNA genes using gene specific primers homologous to 200 bp up and down stream of the tRNA gene which was deleted in the library. Genomic DNA of Y5565 was used to amplify the desired tRNA gene, followed by cloning into the pRS316 centromeric plasmid.

**Creation of multi-deletion tRNA strains**

Double deletion of tRNA genes was constructed in a similar way to the creation of the tRNA deletion library strains. A strain from the tRNA deletion library served as the genetic background for the deletion of an additional gene by means of homologues recombination. The sequential PCR was repeated for the second tRNA gene deletion using the same primers used for the creation of the tRNA deletion library. The templates for the first PCR round were either the pAG25 plasmid containing the nourseothricin N-acetyl-transferase that confers resistance to the antibiotics nourseothricin, or the pFA6a-kanMX6, encoding the kanamycin gene that confers resistance to the antibiotics G418. Triple deletions were created by mating double deletions with a single deletion.

**Growth measurements and normalization**

The measures obtained for each strain in the library were compared to wild-type values that were retrieved separately for each condition. The wild-type measures were done in three repeats and were then summarized by the mean and standard deviation values to generate normalized growth rate and normalized growth yield for each condition. This procedure allowed normalizing for potential variation between days, incubator slots, and locations of wells within the 96-well plate. These measures were obtained from three biological repeats containing wild-type cells in all plate positions, for all slots in the incubator. Each mutant was characterized by growth rate and growth yield. We consider a mutant as showing a phenotype if it deviated by more than two standard deviations from the wild-type mean in that condition in either growth rate or growth yield.

**Double deletion epistasis calculations**

The epistasis between any two deletion strains was calculated according to the non-scaled measure of epistatic interactions [9] using the following equation:

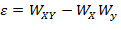
In which
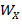
 and
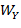
 represent the growth values (separating growth rate and growth yield) of the single deletions and
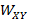
 represents the growth values of the corresponding double deletion. The results of the calculation indicate the nature of the epistasis,
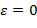
 indicates no epistasis,
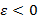
 aggravation and
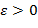
 buffering.

# Note

**No appreciable correlation between tRNA expression and the expression levels of nearby protein-coding genes**

It has been demonstrated in the past (mainly for specific cases) that the expression of tRNA genes may affect the expression levels of nearby coding genes [6–8]. In order to explore a possible relation between the essentially of tRNA genes, as revealed here upon their deletion, and the expression of nearby genes we measured the correlation between the tRNA deletion phenotype and the expression level (as measured for the wild-type strain in our microarray data) of their upstream and downstream nearby genes. We found no correlation between the severity of growth yield or growth rate phenotypes and nearby gene expression level, indicating that high (or low) expression of a nearby gene is not a predictor of fitness change upon tRNA deletion (see Supplemental figure S3A,B and Supplemental table S1). In addition, we did not find any correlation between the distance to the closest genomic feature and the phenotype of the deletion strain.

Furthermore focusing on five tRNA deletions for which we measured genome wide expression changes, we examined the effect of the deletion on the expression levels of the adjacent genes. As can be seen in the following table:

| Deletion Strain | Upstream | | | Downstream | | |
| --- | --- | --- | --- | --- | --- | --- |
|  | Gene Name | Distance bp | log_2_ (Fold Change) | Gene Name | Distance bp | log_2_ (Fold Change) |
| *∆iM(CAU)C* | YCR018C | -1017 | 0.21 | YCR019W | 2846 | 0.05 |
| *∆R(CCU)J* | YJR054W | -1006 | 0.27 | YJR055W | 146 | -0.97 |
| *∆tR(UCU)M2* | YML071C | -253 | -0.37 | YML070W | 1579 | -0.38 |
| *∆tH(GUG)G1* | YGL205W | -221 | 0.79 | YGL203C | 1779 | 0.07 |
| *∆L(GAG)G* | YGR106C | -889 | -0.12 | YGR108W | 2880 | -0.37 |

Out of 10 protein-coding genes examined, only in two cases (*YJR055W* and *YGL205W*) we detect an appreciable change in the expression level (although in both cases it was less than two fold). This indicates that in most cases tRNAs deletion do not affect their surroundings.

The general lack of coordination between tRNA genes and nearby protein-coding genes is in agreement with the work of Conesa *et al*., [9], that have measured the expression level of all protein-coding genes in several Pol III mutants as well as few tRNA deletion strains. This analysis detected only a modest correlation between altered expression of Pol II-transcribed genes and their proximity to class III genes.

For the deletion that demonstrated the highest fold change in the expression of a nearby gene (*tR(CCU)J)* and a severe phenotype, we verified that the fitness reduction observed in this strain is indeed due to the deletion of the tRNA gene using several different methods. First, using complementation assay we re-introduced the *tR(CCU)J* gene on a centromeric plasmid into the background of the deleted strain. This resulted in a complete abolishment of the fitness defect (Supplemental figure S3C). In addition, we measured the growth of a strain deleted for the *YJR055W* gene (the protein-coding gene located downstream *tR(CCU)J)* and found that unlike *tR(CCU)J* this strain does not exhibit a growth rate phenotype (Supplemental figure S3C). Taken together these two analysis demonstrate that the growth defect observed in cells deleted for *tR(CCU)J* is due to the lack of function of the CCU tRNA.

**Supplemental references**

1. Goldstein AL, McCusker JH (1999) Three new dominant drug resistance cassettes for gene disruption in Saccharomyces cerevisiae. Yeast (Chichester, England) 15: 1541–1553. doi:10.1002/(SICI)1097-0061(199910)15:14<1541::AID-YEA476>3.0.CO;2-K.

2. Wach A, Brachat A, Alberti-Segui C, Rebischung C, Philippsen P (1997) Heterologous HIS3 marker and GFP reporter modules for PCR-targeting in Saccharomyces cerevisiae. Yeast (Chichester, England) 13: 1065–1075. doi:10.1002/(SICI)1097-0061(19970915)13:11<1065::AID-YEA159>3.0.CO;2-K.

3. Sikorski RS, Hieter P (1989) A system of shuttle vectors and yeast host strains designed for efficient manipulation of DNA in Saccharomyces cerevisiae. Genetics 122: 19–27.

4. Brachmann CB, Davies A, Cost GJ, Caputo E, Li J, et al. (1998) Designer deletion strains derived from Saccharomyces cerevisiae S288C: a useful set of strains and plasmids for PCR-mediated gene disruption and other applications. Yeast (Chichester, England) 14: 115–132. doi:10.1002/(SICI)1097-0061(19980130)14:2<115::AID-YEA204>3.0.CO;2-2.

5. Elena SF, Lenski RE (1997) Test of synergistic interactions among deleterious mutations in bacteria. Nature 390: 395–398. doi:10.1038/37108.

6. Hull MW, Erickson J, Johnston M, Engelke DR (1994) tRNA genes as transcriptional repressor elements. Molecular and cellular biology 14: 1266–1277.

7. Kendall A, Hull MW, Bertrand E, Good PD, Singer RH, et al. (2000) A CBF5 mutation that disrupts nucleolar localization of early tRNA biosynthesis in yeast also suppresses tRNA gene-mediated transcriptional silencing. Proceedings of the National Academy of Sciences of the United States of America 97: 13108–13113. doi:10.1073/pnas.240454997.

8. Bolton EC, Boeke JD (2003) Transcriptional interactions between yeast tRNA genes, flanking genes and Ty elements: a genomic point of view. Genome research 13: 254–263. doi:10.1101/gr.612203.

9. Conesa C, Ruotolo R, Soularue P, Simms TA, Donze D, et al. (2005) Modulation of yeast genome expression in response to defective RNA polymerase III-dependent transcription. Molecular and cellular biology 25: 8631–8642. doi:10.1128/MCB.25.19.8631-8642.2005.
